# Time Resolved DNA Barcodes for Information Encoding and Dynamic Encryption

**DOI:** 10.1002/advs.76492

**Published:** 2026-07-09

**Authors:** Likang Chu, Haixia Wang, Haiyan Gao, Sixian Chen, Zilong Li, Zhanyihao Hao, Lei He, Da Han

**Affiliations:** ^1^ The Key Laboratory of Zhejiang Province for Aptamers and Theranostics Hangzhou Institute of Medicine (HIM) Chinese Academy of Sciences Hangzhou Zhejiang China; ^2^ University of Chinese Academy of Sciences Beijing China; ^3^ School of Molecular Medicine Hangzhou Institute for Advanced Study University of Chinese Academy of Sciences Hangzhou Zhejiang China; ^4^ College of Materials Science and Engineering Zhejiang University of Technology Hangzhou Zhejiang China; ^5^ College of Pharmaceutical Science Zhejiang University of Technology Hangzhou Zhejiang China; ^6^ Institute of Molecular Medicine (IMM) School of Medicine Renji Hospital Shanghai Jiao Tong University Shanghai China

**Keywords:** DNA nanotechnology, DNA Temporal Barcode, information encoding, information encryption

## Abstract

The rapid expansion of the information era necessitates molecular information encoding systems that simultaneously offer high capacity and robust security. DNA, characterized by its ultra‐high information density, outstanding chemical stability, and inherent programmability, stands out as a promising medium for molecular data storage. Nevertheless, traditional DNA sequence‐based information encoding strategy remains intrinsically static, restricting real‐time and dynamic manipulation of stored information, and thereby limiting its practical utility in adaptive data processing and dynamic encryption. To overcome these limitations, we present a programmable data encoding and dynamic encryption platform leveraging DNA Temporal Barcodes (DTBs). DTBs consist of DNA strands designed with distinct retention times during high‐performance liquid chromatography (HPLC) separation. Through systematic programming of chemical modifications and DNA sequences, we constructed a versatile DTB library capable of encoding a broad diversity of information states. HPLC, while providing time‐resolved readout for DTBs, also allows for their efficient recovery and subsequent reuse in information encoding. Notably, to further reinforce data security, we introduce a key‐triggered DNA ligation mechanism that generates reconfigurable DTBs, facilitating dynamic encryption at the molecular level. This work establishes a versatile strategy for constructing programmable, high‐capacity, and dynamically adaptable molecular information security systems.

## Graphical abstract

This study establishes a molecular information platform based on DNA Temporal Barcodes. Information is encoded through combinations of DNA tags with distinct retention times, while dynamic encryption is achieved through a key‐triggered DNA ligation. In addition, high‐performance liquid chromatography (HPLC) enables high‐fidelity decoding and tag recovery, providing a strategy for high‐capacity, reconfigurable molecular information security.

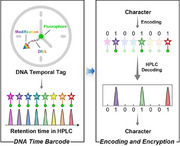

## 1 Introduction

The digital revolution has fundamentally redefined how information is generated, transmitted, and archived, yet contemporary electronic cryptographic infrastructures continue to face inherent structural constraints. Most prominently, widely deployed schemes are grounded in a finite set of mathematically hard problems and associated computational hardness assumptions, so that their long‐term robustness is inextricably tied to prevailing models of classical and quantum computation [1]. As algorithmic advances and scalable quantum architectures emerge, these assumptions may be progressively eroded, exposing current systems to both prospective compromise and retrospective decryption of data presumed secure at the time of storage. In parallel, digital information is predominantly represented as readily duplicable electronic signals that traverse densely interconnected networks, creating an attack surface that is intrinsically amenable to automation and large‐scale orchestration. Adversaries can systematically interrogate software stacks, hardware implementations, and communication protocols, exploiting even subtle implementation flaws at negligible marginal cost [2]. Furthermore, existing cryptographic mechanisms are almost entirely confined to the electronic domain and lack native means to interface with biochemical processes, thereby limiting their applicability in emerging life science and bioengineering contexts where secure communication, actuation, and decision‐making must occur within or across living systems [3]. Collectively, these factors motivate the search for alternative information paradigms that decouple security from purely algorithmic hardness, introduce substantial physical and experimental barriers to adversarial access, and span the boundary between digital and biological domains.

Against this backdrop, molecular information systems have emerged as a compelling complementary framework. Rather than encoding data exclusively as abstract binary symbols, molecular approaches embed information directly into the intrinsic physicochemical properties, sequences, and state configurations of chemical and biological substrates [4, 5]. By harnessing highly specific molecular recognition events, programmable chemical modification patterns, and stimuli‐responsive architectures, such systems can implement encryption schemes that are both structurally rich and intrinsically concealed within complex material matrices. Unauthorized decryption typically necessitates sophisticated wet‐lab protocols and advanced analytical platforms, such as high‐resolution spectroscopy, chromatography, or sequencing, thereby replacing the low‐cost, fully automated nature of conventional cyberattacks with resource‐intensive experimental efforts [6, 7]. In addition, molecular carriers can be rationally engineered to undergo dynamic, reversible, or irreversible transformations in response to external cues including light, temperature, pH, redox state, or defined biomolecular signals [8]. This enables time‐gated, environment‐dependent, or self‐erasing modes of access control that operate at the level of physical matter, and which are exceedingly difficult to reproduce within standard electronic hardware [9]. Critically, when constructed from nucleic acids, proteins, or other biocompatible polymers, molecular encryption schemes can be directly wired into cellular signaling pathways, gene regulatory circuits, and metabolic networks, thereby supporting secure information processing and decision‐making within living systems themselves [10]. Taken together, these attributes position molecular information systems not merely as a niche alternative, but as a qualitatively distinct security layer that augments traditional cryptography in scenarios demanding physical steganography, experimentally enforced access constraints, and deep integration with life‐science platforms.

DNA stands out as a highly attractive medium for molecular data storage due to its extraordinary information density, exceptional chemical stability, and inherent programmability [11, 12]. Significant advances in DNA‐based information encoding have enabled the storage of massive quantities of data within DNA sequences, achieving substantial gains in both storage density and longevity compared to conventional media [13, 14, 15]. However, a critical limitation of this strategy lies in the intrinsic static nature of DNA—once the DNA sequences are synthesized, they are exceedingly challenging to modify or update. This poses considerable challenges for scenarios requiring frequent data revision or dynamic encryption, thereby limiting the versatility and responsiveness of DNA‐based information systems in such contexts [16]. In contrast, non‐sequence DNA information encoding systems have emerged as important complements to sequence‐based DNA storage. These systems do not rely solely on nucleotide order, but instead encode information into readable features generated by DNA‐based constructs, such as micro‐/nanostructural patterns, fluorescence signals, mass‐spectrometric signatures, or electrical responses. For instance, DNA self‐assembled structures enable information storage and encryption through fluorescence–spatial distributions or dynamic conformational changes [17]; mass encoding uses characteristic mass‐to‐charge signals from DNA nanodevices to construct molecular barcodes [18]; and nanopore‐current encoding converts molecular binding events on linear DNA scaffolds into characteristic current‐blockade signals [19]. Although these strategies have expanded the encoding dimensions and dynamic capabilities of DNA‐based molecular information systems, it remains challenging to integrate high coding density, automated readout, dynamic programmability, secure encryption, and carrier reuse within a single non‐sequence DNA encoding framework.

Biological systems typically operate with a limited repertoire of signaling molecules, yet they are capable of encoding highly complex and specific information by precisely modulating the temporal dynamics of these signals [20, 21]. This fundamental observation motivates a central question: Can artificial molecular information systems likewise harness the temporal dimension to expand their effective encoding capacity? High‐performance liquid chromatography (HPLC) presents a distinct advantage over platforms like mass spectrometry. It inherently provides time‐resolved readout, establishing a natural physical framework for time‐domain encoding strategies [22]. Furthermore, a key benefit is the ability to recover these information‐encoded labels, paving the way for their repeated and sustainable use. When coupled with fluorescence detection, HPLC separates different molecular species into discrete chromatographic peaks at well‐defined retention times, enabling multiplexed discrimination of fluorescent signals along the time axis. In this configuration, even signals that exhibit substantial spectral overlap can be reliably resolved on the basis of their elution times, thereby substantially mitigating the constraints imposed by spectral congestion in purely fluorescence‐based schemes and, in turn, enhancing the overall information encoding capacity of the system [23].

Building on these considerations, we harness the time‐resolving power of HPLC and the molecular programmability of DNA to establish a scalable information encoding and dynamic encryption platform based on DNA Temporal Barcodes (DTBs). We exploit the fact that single‐stranded DNA exhibits precisely tunable HPLC retention times as a joint function of its chemical modifications and sequence composition, and systematically identify eight mutually orthogonal strands with non‐overlapping retention windows under a single HPLC method. Extending this design to two independent detection channels, the combinatorial use of these orthogonal strands yields a theoretical library of 65,536 distinct DTB states. By assigning a binary value to each DTB—“1” for presence and “0” for absence—and encoding text using the American Standard Code for Information Interchange (ASCII) scheme, we demonstrate reliable digital information storage, thereby highlighting the high‐capacity potential of this temporal molecular encoding strategy. HPLC‐based readout further affords a highly automated, high‐fidelity decoding process with 100% accuracy, underscoring the practical feasibility of the approach for molecular data storage. To endow the platform with dynamic programmability, we additionally engineer DTBs that respond to biologically relevant triggers, such as enzymatic reactions, thereby rendering successful decoding contingent on both the temporal barcode and specific molecular cues. This dual‐layer encryption scheme markedly enhances system security and establishes a conceptual bridge between information science and molecular biology, illustrating the transformative potential of integrating temporal and molecular logic in next‐generation data systems.

## 2 Results and Discussion

### 2.1 Design and Optimization of DNA Temporal Tags

**FIGURE 1:**
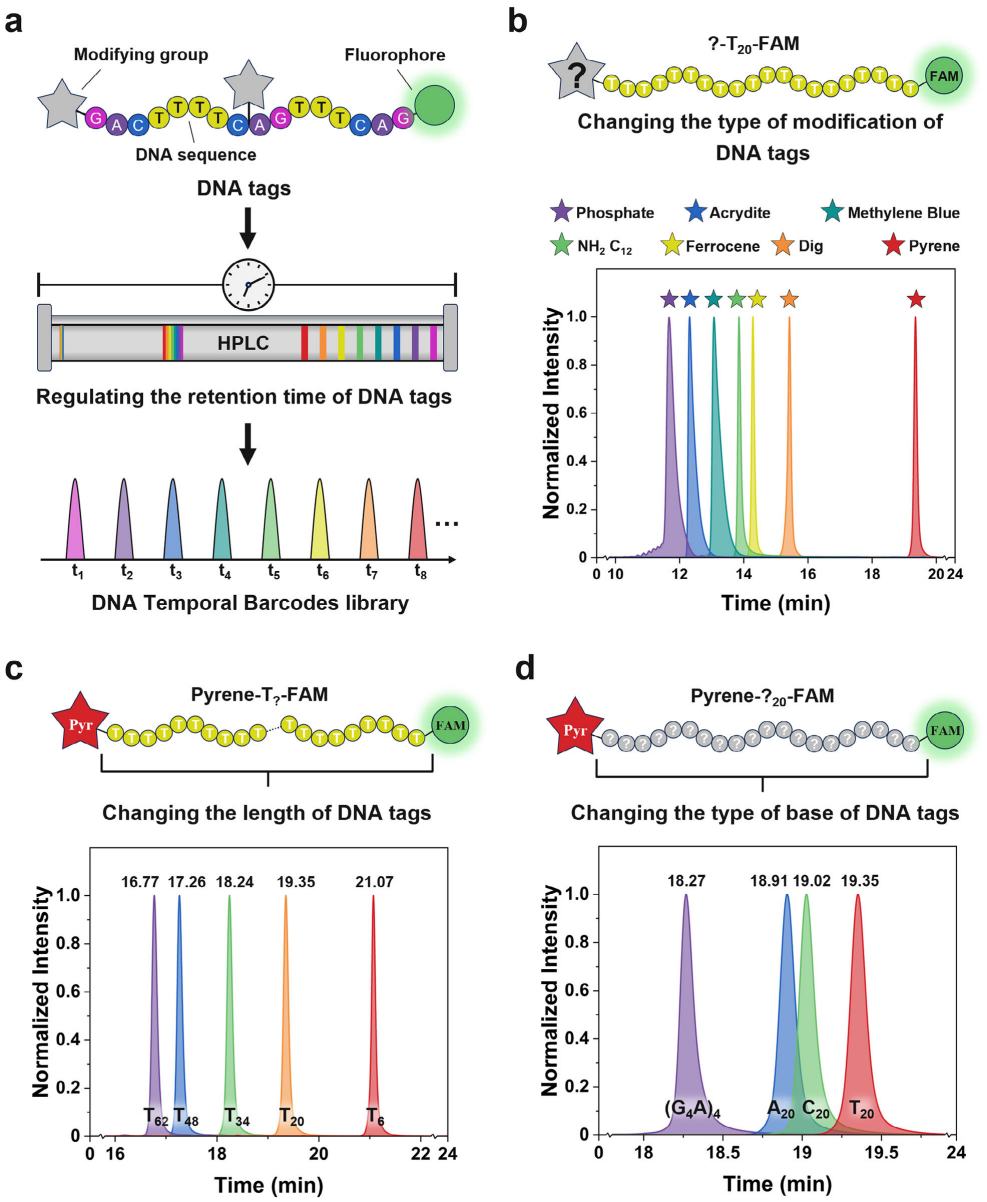
Design of DNA tags and investigation of factors affecting their retention time in HPLC. (a) Retention times of DNA tags can be flexibly tuned by modifying chemical groups and DNA sequence features. (b) Effect of different 5′ modifications on DNA tags retention time. (c) Effect of sequence length (nucleotide count) on retention time. (d) Effect of base type on retention time. For guanine‐rich sequences, a small number of adenines were inserted as spacers to avoid repetitive guanine‐induced synthesis challenges. Fluorescence signals in all HPLC chromatograms were normalized.

To construct DNA Temporal Barcodes (DTBs), it is first necessary to obtain a set of DNA tag molecules that possess highly orthogonal and non‐overlapping retention times during HPLC analysis. The diverse combinations of these orthogonal DNA tags provide a robust foundation for establishing a large DTB library. As illustrated in Figure 1a, by varying the chemical modifications and sequence of DNA molecules, we aimed to systematically modulate their retention times in HPLC, thereby enabling the selection and optimization of ideal orthogonal DNA tags. To ensure efficient discrimination among different DNA tags, all chromatographic analyses were performed under standardized ion‐pair reversed‐phase HPLC conditions using a C18 column with triethylammonium acetate (TEAA)/acetonitrile gradient elution at 60°C. As a first step, we examined how chemical modifications and DNA sequence affect DNA tag's behavior in HPLC. Specifically, to assess the impact of different chemical modifications, we utilized a homopolymeric thymidine DNA sequence (T20) bearing a 3'‐terminal 6‐carboxyfluorescein (FAM) label as the backbone and introduced various modifications at the 5' end (see structures in Figure S1). The resulting DNA tags exhibited distinct retention times in HPLC, ranging from 11 to 20 min depending on the type of modification. The retention times increased in the following order: phosphate (P), acrydite (Acr), methylene blue (Mb), amino‐C12 (NH_2_), ferrocene (Fer), digoxigenin (Dig), and pyrene (Pyr). In general, hydrophilic modifications such as phosphate reduced retention time, whereas hydrophobic groups like pyrene significantly prolonged it (Figure 1b). To expand the variety of programmable DNA tags, we further introduced additional groups, including hydroxyl (OH), biotin (Bio), dibenzocyclooctyne (DBCO), and octadecyl chains (C_18_), which further expanded the retention time window (Figure S2). On the other hand, the number of chemical modifications was also found to affect the retention behavior of DNA tags. For instance, introducing two hydrophobic groups at the 5' end further increased the retention time Figure S3a). In line with previous studies showing that hydrophobic interactions between DNA bases and the stationary phase are position‐dependent—with terminal bases exhibiting stronger interactions—we further demonstrated that the spatial position of the modification is crucial [24]. Specifically, shifting the modification from the 5' terminus to an internal region of the DNA tag resulted in a marked decrease in retention time (Figure S3b).

After establishing that chemical modifications significantly affect the retention time of DNA tags, we next examined the impact of DNA sequence features—specifically sequence length and base identity—on retention times. To investigate the effect of sequence length, we synthesized DNA tags composed entirely of thymidines (T) with varying lengths and analyzed their retention times using HPLC. As shown in Figure 1c, DNA tags modified with 5'‐pyrene exhibited progressively shorter retention times as the sequence length increased. The elution‐advancing effect diminished with increasing length, as evidenced by the decreasing peak‐to‐peak spacing. A similar trend was observed with other chemical modifications, such as 5'‐ferrocene, indicating a generalizable phenomenon (Figure S4). This trend is likely attributable to stronger electrostatic interactions between longer single‐stranded DNA (ssDNA) and ion‐pairing reagents, arising from the greater number of negatively charged phosphate groups under neutral conditions, which facilitate earlier elution [25]. As the DNA length increases, the relative charge difference between DNA tags of varying lengths diminishes, thereby reducing chromatographic resolution. We further explored whether the type of DNA base affects the retention time of DNA tags, as the bases in ssDNA are directly exposed to the mobile phase. To evaluate this, we synthesized a series of 20‐nt homopolymeric DNA tags composed of adenine (A), thymine (T), cytosine (C), or guanine (G; with minor A spacers to facilitate synthesis), each modified with a 5'‐pyrene and 3′‐FAM, and analyzed them by HPLC. As shown in Figure 1d, the retention times followed the order G < A < C < T, likely reflecting differences in base hydrophobicity. Overall, base identity had a modest impact on retention time, with differences of up to 1.5 min, consistent with the relatively small hydrophobic variation among nucleobases. Therefore, three key factors were identified as governing the retention time of DNA tags in HPLC: chemical modifications exerted the strongest influence, modulating retention times by up to approximately 15 min (Figure 1b and Figure S2); sequence length had a moderate impact, typically causing shifts of 4–5 min (Figure 1c); and base identity showed a relatively minor effect, with differences within roughly 1.5 min (Figure 1d).

### 2.2 Construction of DNA Temporal Barcodes for Information Encoding

**FIGURE 2:**
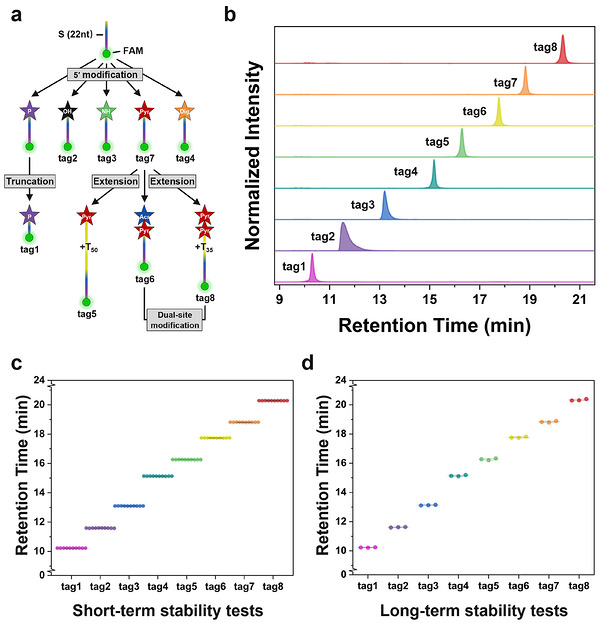
Selection and retention time stability assessment of DNA Temporal Barcodes for information encoding. (a) Design workflow of FAM‐labeled DNA tags with distinguishable retention times in HPLC. A 22‐nt ss DNA (denoted as S), labeled with FAM at the 3′ end, was used as the scaffold. Various chemical groups—including phosphate (P), hydroxyl (OH), acrydite (Acr), amino‐C12 (NH_2_), digoxigenin (Dig), and pyrene (Pyr)—were introduced at the 5′ end of S to generate DNA tags with distinct retention time (e.g., tag1, tag2, tag3, tag4, and tag7). To further increase the diversity of resolvable DTBs, dual‐site modifications were introduced at different positions on S (e.g., tag6 and tag8). Sequence parameters, including strand length and base composition, were also adjusted to optimize resolution. For example, tag1 was obtained by truncating S, while tag5 and tag8 were generated by extending S with poly‐T at the 5′ end. (b) Vertically offset HPLC chromatograms obtained from separate injections of eight individual FAM‐labeled DNA tags. The x‐axis represents retention time, and the y‐axis represents normalized FAM fluorescence intensity. (c) Short‐term reproducibility test of retention times for FAM‐labeled DTB library, assessed by ten consecutive HPLC injections of a mixture containing all eight DNA tags. (d) Long‐term reproducibility test of retention times for FAM‐labeled DTB library over 47 days, assessed by HPLC analysis of a mixture containing all eight DNA tags on Day1,Day40 and Day47. Each DNA tag was dissolved in diethyl pyrocarbonate (DEPC)‐treated water to prepare a 20 µM stock solution and stored at 4°C before HPLC analysis. Black horizontal lines across the data points represent the mean retention times of repeated measurements; error bars indicate mean ± 1 standard deviation.

After establishing the temporal programmability of DNA tags, we further explored the construction of high‐resolution and diverse DTBs by designing and combining DNA tags with predictable retention time trends. These DTBs were then utilized for molecular information encoding. As shown in Figure 2a, we systematically optimized key parameters influencing the retention time of DNA tags—including the type, number, and position of chemical modifications, as well as DNA length and base composition—and identified eight FAM‐labeled DNA tags with clearly distinguishable retention times. These DNA tags produced sharp, well‐resolved, and non‐overlapping peaks in the HPLC chromatograms (Figure 2b), thereby allowing for reliable peak assignment during decoding. According to their retention time order, these were designated as DNA tags 1 through 8, forming the foundation of the DTB library. In theory, eight orthogonal DNA tags can provide an encoding space of 2^8^ unique combinations, thereby establishing a scalable basis for molecular data storage and encryption.

To further evaluate the stability of the retention times of DNA tag 1 through tag 8 comprising the DTBs, we next focused on two key performance indicators: orthogonality and reproducibility. For orthogonality assessment, we simultaneously injected a mixture of the eight FAM‐labeled DNA tags and analyzed them using HPLC (Figure S5), comparing their retention times to those obtained from individual injections (Figure 2b and Table S1). The results demonstrated highly consistent retention times with a maximum deviation of no more than 0.09 min, indicating negligible signal interference between tags and thus excellent chromatographic orthogonality. For reproducibility evaluation, we conducted both short‐term and long‐term stability tests of the retention times. In the short‐term assessment, the eight FAM‐labeled DNA tags were subjected to ten consecutive HPLC runs (Figure 2c), yielding highly consistent retention times across all tags, with coefficients of variation (CVs) below 0.07%. This confirms the high precision and suitability of our method for high‐throughput decoding (Table S2). In the long‐term analysis, three independent HPLC runs using different sample batches were performed over a 47‐day period (Figure 2d). Despite the extended timespan, retention times remained highly stable, with all CVs below 0.4%, further demonstrating the feasibility of this system for long‐term data storage applications (Table S3).

### 2.3 Information Encoding System Based on DNA Temporal Barcodes

**FIGURE 3:**
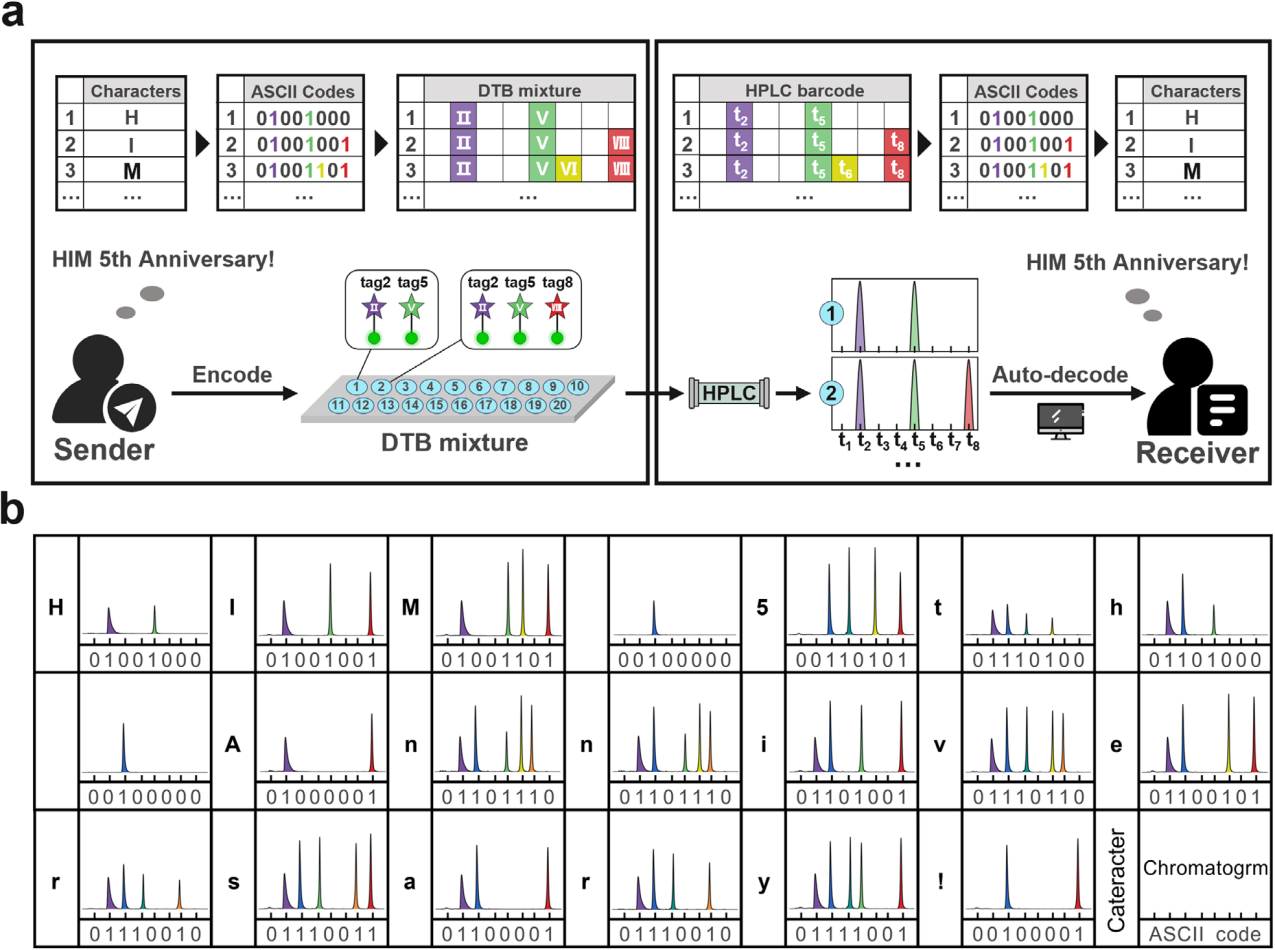
Construction and application of time‐resolved information encoding system. (a) Schematic of the encoding system using DTBs for communication. (b) HPLC‐based decoding of information stored in DTBs mixtures. The x‐axis denotes retention time (9–21.7 min), with tick marks indicating the retention times of FAM‐labeled DNA tag1–tag8. The y‐axis represents fluorescence intensity.

After selecting a set of DNA tags with well‐defined retention times to construct the DTB library, we proceeded to establish a molecular information encoding system based on DTBs and evaluate its effectiveness for molecular‐scale data storage and transmission. Specifically, this system utilizes eight FAM‐labeled DNA tags as molecular carriers to encode information by mapping all 256 possible DTB combinations onto the ASCII character set, allowing the representation of a wide range of characters—including uppercase and lowercase letters, numbers, and punctuation marks—each encoded as an 8‐bit binary string [26]. Information decoding is then achieved by interpreting the unique HPLC output profiles. To ensure the accuracy and standardization of information processing, we established a detailed protocol defining the encoding and decoding workflows. During the encoding phase, each character is first converted into an 8‐bit binary sequence according to the ASCII table. Each bit is mapped to the presence (“1”) or absence (“0”) of the corresponding DNA tag (tag1–tag8), resulting in a unique DTB mixture for each character. In the decoding phase, each encoded sample is analyzed using standardized HPLC procedures (see Methods). Signal peaks that appear within eight predefined retention time windows are utilized to reconstruct the binary sequence, which is subsequently decoded into the original character according to the ASCII table (Table S4 and Table S5). As a demonstration, we applied this time‐resolved encoding system for the molecular storage and transmission of the message “HIM 5th Anniversary!” (Figure 3a). The specific process is as follows: (1) Encoding: The 20 characters comprising the message were individually converted into 20 lines of 8‐bit ASCII binary code, yielding a total information content of 160 bits. Each binary string was then used to generate a specific DTB mixture following the established encoding protocol; for example, the letter “H” (01001000) corresponds to a mixture containing only DNA tag2 and DNA tag5. (2) Storage and transmission: The 20 encoded samples were sequentially labeled and stored under appropriate conditions. For transmission, the physically labeled samples were delivered to the recipient. (3) Decoding: The recipient sequentially performed HPLC analysis on the 20 samples. The chromatograms for each sample were used to extract the binary sequence based on the appearance of signal peaks in the eight defined retention time windows, and the resulting binary codes were decoded into the original characters using the ASCII table. For instance, the first sample exhibited peaks only at the retention times corresponding to DNA tag2 and DNA tag5, yielding the binary sequence “01001000,” which decodes to “H.” Ultimately, the decoded output was found to be identical to the original message, thus demonstrating the high fidelity and reliability of the encoding and decoding system (Figure 3b). In addition, we have developed an initial program that directly converts raw HPLC outputs into readable information, substantially simplifying the decoding workflow (Figure S6).

### 2.4 Dynamic Information Encryption System Based on Target‐Induced Reconstruction of DNA Temporal Barcodes

**FIGURE 4:**
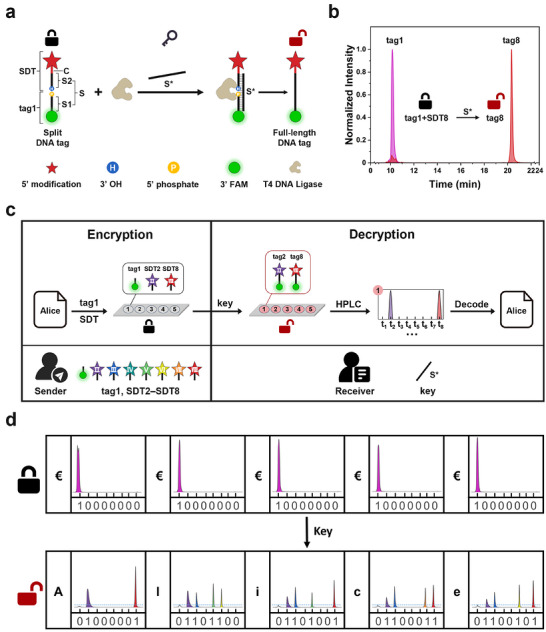
Construction and application of dynamic information encryption system. (a) Principle of the dynamic encryption strategy based on enzyme‐mediated DNA ligation. (b) Validation of the encryption mechanism, illustrated using tag1 and SDT8 as an example. (c) Implementation of dynamic encryption for secure information transmission. (d) HPLC decoding of encrypted samples before and after decryption. In each chromatogram, the x‐axis denotes retention time (9–21.7 min), and the y‐axis denotes FAM fluorescence intensity. The blue dashed line marks the decoding threshold used to exclude low‐intensity residual tag1 signals caused by incomplete ligation: intensities above the threshold are decoded as “1”; otherwise, it is decoded as “0”.

Although the DTB‐based encoding system provides a relatively high level of security, the encoded information remains susceptible to compromise if the decoding protocol is disclosed [27]. To further enhance system security, we developed a dynamic encryption strategy based on enzyme‐mediated DNA ligation (Figure 4a) [28]. In this approach, the structure of the DNA tags was rationally modified to enable ligation‐mediated information reconstruction. Specifically, DNA tag1 remained intact, while DNA tag2 through DNA tag8 were cleaved at the midpoint of their DNA scaffold (S) to generate two fragments: one fragment, termed Split DNA Tag (SDT), and the other corresponding to DNA tag1. According to the established encoding protocol, each character was encrypted as a mixture of DNA tag1 and the appropriate SDT (SDT2–SDT8 replacing DNA tag2–DNA tag8). Importantly, the SDTs lack FAM labeling and are therefore undetectable by the HPLC fluorescence detector. In the encrypted state, only the signal from DNA tag1 is observed in the chromatogram, and the stored information remains completely concealed. To decrypt the information, a complementary DNA strand (S^*^) is introduced as the decryption key. Because DNA tag1 and the respective SDTs contain different segments of S, DNA strand S^*^ can hybridize with both to form a ternary complex. In the presence of T4 DNA ligase, a phosphodiester bond is formed between the 5′‐phosphate of DNA tag1 and the 3′‐hydroxyl of the SDT, reconstituting the full‐length DNA tag. The regenerated DNA tag yields a distinct retention peak in the HPLC chromatogram, thereby enabling information retrieval.

To further validate this encryption mechanism, DNA ligation was performed between tag1 and each of SDT2–SDT8 individually in the presence of T4 DNA ligase, and HPLC analyses were performed before and after the addition of DNA strand S^*^ to characterize the different information states (Figure 4b and Figure S7). Prior to the addition of DNA strand S^*^, the chromatogram displayed a single peak corresponding to DNA tag1, with no evidence of ligation products, indicating that the information was securely encrypted. After the introduction of DNA strand S^*^, the DNA tag1 peak diminished markedly, while new peaks appeared at the retention times corresponding to the target DNA tags. This result confirms that, upon enzymatic activation, SDT2–SDT8 efficiently ligate with DNA tag1 to reconstitute the full‐length DNA tags, thereby enabling the decryption of the encoded information. Notably, the retention times of the ligation products were highly consistent with those of the original full‐length DNA tags, indicating effective dissociation of the restored DNA tags from DNA strand S^*^ under the 60°C HPLC conditions with TEAA/acetonitrile gradient elution. This guarantees accurate time‐resolved decoding following enzymatic activation. We also observed that a weak residual DNA tag1 peak remained after ligation, likely due to incomplete conversion under the current reaction conditions. Therefore, a signal‐intensity decoding threshold was applied in subsequent analysis to exclude this residual signal and avoid false‐positive binary assignment.

We further implemented this dynamic encryption mechanism for secure communication. In this system, DNA tag1 and SDT2–SDT8 serve as the encryption‐encoding components. These molecular components, together with the encoding rule, may be distributed to the information sender; however, their exact nucleotide sequences should not be publicly disclosed. In contrast, the decryption key S^*^, the ligation‐based decryption procedure, and the HPLC decoding rule are retained only by the recipient to ensure information security. As a proof of concept, we encrypted and successfully retrieved the message “Alice” through the following process (Figure 4c): (1)Encryption: the sender encoded the five‐character message according to the established encoding rule, generating five separate hybrid samples, each containing DNA tag1 and specific SDTs, and sent them sequentially to the recipient; (2)Decryption: upon receipt, the recipient added the key S^*^ to initiate the DNA ligation reaction catalyzed by T4 DNA ligase, followed by HPLC analysis. As shown in Figure 4d, the resulting chromatograms were decoded according to the decoding rule, and the original message was fully recovered with 100% accuracy using a predefined signal intensity threshold to exclude residual tag1 signals. It is noteworthy that if the encrypted samples were intercepted during transmission, an eavesdropper lacking the key S^*^ would only detect the DNA tag1 signal under the HPLC readout workflow; consequently, all samples would be decoded as “10000000,” corresponding to an irrelevant character (“€”), thus preventing information leakage. In summary, this dynamic encryption system introduces a biological trigger into the decoding process, requiring not only knowledge of retention time patterns but also access to a sequence‐specific molecular key for information retrieval. This dual‐layer security mechanism markedly enhances both the safety and robustness of molecular data storage and communication.

## 3 Discussion

In summary, we have developed a new class of DNA‐based information materials, termed DTBs, and established a molecular information platform that integrates both information storage and dynamic encryption capabilities. Through the systematic investigation of key factors affecting DNA tag retention time in high‐performance liquid chromatography (HPLC), including chemical modifications and sequence design, we achieved precise temporal programmability of DNA tags. On this basis, we constructed a barcode library in which a single fluorescence channel accommodates eight DNA tags with distinct retention times, resulting in a theoretical encoding capacity of up to 2^8^ = 256 unique combinations. Furthermore, the stability of DNA tag retention times across different experimental batches, together with the use of predefined retention time windows for individual tag decoding rather than exact retention time values, provides tolerance to minor chromatographic drift. Using this system, we successfully demonstrated high‐fidelity molecular storage and decoding of digital information.

Beyond static information encoding, we further introduced a key‐triggered DNA ligation mechanism to construct a dynamic encryption architecture. This strategy greatly enhances data security and provides a new conceptual framework for the integration of molecular informatics with functional biological components. To the best of our knowledge, this work represents the first demonstration of HPLC‐mediated time‐domain DNA information encoding, offering a complementary strategy that addresses the static limitations of conventional sequence‐based DNA storage and enabling programmable and dynamic encryption. These advances not only drive progress in the fields of DNA data storage and molecular cryptography but also lay a solid foundation for next‐generation temporally programmable molecular information systems.

To further expand the encoding capacity, we employed a fluorescence channel multiplexing strategy by screening an additional set of DNA tags labeled with the Alexa Fluor 647 (AF647) fluorophore (Figure S8). These AF647‐labeled DNA tags were specifically designed to closely align with the retention profiles of their FAM‐labeled counterparts, thereby enabling dual‐channel synchronous detection (Figure 2b and Figure S8). Under this dual‐channel detection scheme, each temporal window theoretically accommodates four distinct signal states (absence of signal, FAM‐signal exclusively, AF647‐signal exclusively, or simultaneous presence of both signals), thereby augmenting the total encoding combinations from 2^8^ = 256 to 4^8^ = 65 536. These results suggest that the integration of fluorescence multiplexing can further significantly increase the encoding capacity, thereby providing a solid foundation for large‐scale molecular data storage and encryption.

Moreover, unlike destructive readout methods such as mass spectrometry, HPLC provides a nondestructive analytical route, facilitating repetitive information decoding. To validate this, we performed three consecutive HPLC analyses of DNA tags encoding “NTP”, each consistently yielding a clear and accurate decoding result (Figure S9a,c). Beyond nondestructive readout, HPLC's high‐resolution separation enables peak‐by‐peak recovery of each DNA tag after decoding for reuse. Specifically, we collected and recombined the tags decoded from “DEU” and successfully re‐encoded and decoded a new message, “QAT”, demonstrating information rewriting (Figure S9b,d). Collectively, this HPLC‐centered “nondestructive readout–efficient recovery” loop enables multi‐round read–write cycles, supports iterative retrieval and on‐demand updates during information storage and transmission, and elevates HPLC from a passive readout method to an active information‐processing platform capable of iterative operations—offering an engineering pathway toward complex, interactive molecular information systems.

In the DTB system, each DNA tag carries 1 bit of information. Given an average tag length of 28 bases, the logical coding density is 0.036 bits/base, lower than the 1.5–1.8 bits/base reported for sequence‐based DNA encoding systems such as DNA Fountain [29]. However, this lower density is accompanied by enhanced programmability of the encoding units, enabling molecular‐key‐responsive dynamic encryption. Moreover, unlike Illumina sequencing commonly used for sequence‐based DNA storage, HPLC provides non‐destructive analysis coupled with practical DNA tag recovery, making the DTB system suitable for repeated access and dynamic updating rather than only static archival storage. In addition, a detailed comparison with other non‐sequence DNA storage strategies, including structure‐based encoding, mass‐tag encoding, and nanopore‐current encoding, is provided in Table S6.

At the current stage, although the HPLC readout speed remains relatively low (0.33 bits/min; Table S6), HPLC remains a mature and practical readout platform for the DTB system. It simultaneously supports time‐resolved decoding, broad compatibility with chemically modified DNA, stable and automated readout, multidimensional detection expansion, and sample recovery. In the future, capillary electrophoresis and other high‐efficiency separation methods may be explored as alternative readout platforms to reduce analysis time and cost [30].

The security of the DTB dynamic encryption system still has certain boundaries. The current system mainly relies on the confidentiality of the S^*^ sequence. If an adversary obtains the sequences of DNA tag1 and SDT2–SDT8, for example by sequencing, S^*^ could be reverse‐engineered based on Watson–Crick complementarity. Future designs could mitigate this risk by introducing multi‐factor molecular triggering mechanisms, such as controllable terminal activation, so that the encryption components become ligatable only after an additional enzymatic, photochemical, or strand‐displacement trigger. In addition, template‐independent ligases, such as T4 RNA ligase, may allow non‐specific ligation between DNA tag1 and SDTs without S^*^, potentially enabling unauthorized information recovery [31]. To reduce this risk, decoy DNA strands could be introduced as molecular camouflage components. These decoy strands would be compatible with ligation but have sequences distinct from the SDTs. Under S^*^, S^*^ would direct sequence‐specific ligation between DNA tag1 and the target SDTs to generate the correct DTB; in contrast, without S^*^, template‐independent ligation could produce complex mixed products through the random ligation of DNA tag1 with SDTs or decoy strands, generating interfering or false‐encoding peaks in HPLC and thereby increasing the difficulty of unauthorized decryption.

Looking ahead, the encoding capacity of this platform could be further expanded by incorporating additional fluorescence channels and orthogonal chemical modifications [32, 33]. The integration of artificial intelligence‐assisted peak recognition algorithms is expected to accelerate decoding, enhance robustness, and enable fully automated data analysis [34]. Moreover, the incorporation of diverse biomolecular logic and responsive elements will further enrich the system's dynamic encryption capabilities and functional complexity [35, 36].

## 4 Experimental Section

### 4.1 Instruments

DNA concentrations were determined using a Nano‐400A micro‐volume nucleic acid analyzer (Allsheng). HPLC analyses were performed on an Agilent 1260 II system equipped with a reversed‐phase C18 column (InfinityLab Poroshell 120 EC‐C18, 3.0 × 150 mm, 2.7 µm particle size). The pH of the mobile phase was adjusted with a benchtop pH meter (Lanjieke). Samples were concentrated using a ZLS‐1 vacuum centrifugal concentrator (Herexi).

### 4.2 Reagents and Consumables

All DNA oligonucleotides were synthesized by Sangon Biotech and purified by HPLC, with detailed sequences listed in Tables S7–S9. Acetonitrile (HPLC grade; J&K Scientific), acetic acid (analytical grade; Shanghai Hushi), triethylamine (analytical grade; Aladdin), DEPC‐treated water (Sangon Biotech), T4 DNA ligase with its 10× reaction buffer (New England Biolabs), and ultra centrifugal filter (3 kDa molecular weight cutoff; Millipore) were used.

### 4.3 Preparation of HPLC Mobile Phases

Mobile phase A (0.1 M triethylammonium acetate, TEAA) was prepared as follows: to prepare 500 mL of 0.1 M TEAA solution, 2.85 mL of acetic acid was first added to 490.2 mL of ultrapure water and mixed thoroughly. Then, 6.95 mL of triethylamine was added slowly with continued mixing to bring the final volume to 500 mL. The pH was adjusted to 7.0 ± 0.1 using either acetic acid or triethylamine. Before use, mobile phase A was filtered through a 0.22 µm membrane (Jinteng) and degassed by ultrasonic treatment for several minutes. Mobile phase B consisted of HPLC‐grade acetonitrile and was used directly after brief degassing by ultrasonication.

### 4.4 Preparation of HPLC Samples

All DNA samples were dissolved in DEPC‐treated water to a stock concentration of 20 µM and stored at either 4°C or −20°C. Before analysis, the stock solution was diluted with mobile phase A to a final concentration of 100 or 300 nM for FAM‐labeled DNA tags and 400 nM for AF647‐labeled DNA tags. The final sample volume was 120 µL. Samples were then transferred to 300 µL amber autosampler vials (Relab), ensuring no air bubbles remained before injection.

### 4.5 HPLC Analysis Conditions

(1) System preparation: Prior to analysis, both mobile phases A and B were degassed. The column temperature was set to 60°C, and the system was equilibrated under initial conditions (95% A/5% B, flow rate 0.3 mL/min) until temperature and pressure stabilized. (2) Analysis parameters: The injection volume was 100 µL, and the column temperature was maintained at 60°C. Samples were analyzed using a gradient elution method at a constant flow rate of 0.3 mL/min. The gradient program was as follows: 95% A/5% B at 0 min; a linear change to 55% A/45% B from 0 to 20 min; a further linear change to 5% A/95% B from 20 to 22 min; this composition was maintained from 22 to 24 min or longer. A 5.5 min post‐run was included to re‐equilibrate the system to the initial mobile phase composition and pressure, ensuring stability for subsequent sample injections. (3) Fluorescence detection: Signals from DNA tags were detected using a fluorescence detector. For FAM‐labeled DNA tags, the excitation and emission wavelengths were set at 488 and 519 nm, respectively. For AF647‐labeled DNA tags, excitation was set at 642 nm and emission at 672 nm. (4) Maintenance: After all sample runs, the column was flushed with 90% ultrapure water/10% acetonitrile to remove residual buffer salts, followed by 100% acetonitrile for column storage and preservation. (5) HPLC data processing: Chromatographic data were exported from the Agilent 1260 II system and subsequently processed using Origin software for data analysis and chromatogram plotting.

### 4.6 Design and Screening of DNA Temporal Barcodes for Information Encoding

The initial sequences and modifications of DNA tags were preliminarily designed, followed by sequence validation and optimization using NUPACK. By adjusting the sequences, each DNA tag was ensured to adopt a stable single‐stranded conformation under experimental conditions, thereby minimizing the influence of secondary structure formation or intermolecular interactions on its HPLC retention behavior. The designed DNA tags were then synthesized by a commercial provider and evaluated using a standardized HPLC method. Based on the experimental results and established retention time modulation strategies, further structural optimization was performed until DNA tags with retention times within the target range were successfully identified.

### 4.7 DNA Ligation Reaction

Each DNA ligation reaction was performed in a total volume of 20 µL, containing 2 µL of 10× T4 DNA Ligase Reaction Buffer, 2.4 µL of DNA tag1 (10 µM), 2.88 µL of an SDTs mixture (10 µM; selected according to the target character), and 2 µL of T4 DNA Ligase (400 U/µL). The remaining volume was adjusted to 20 µL with DEPC‐treated water. To initiate the ligation reaction, 2.64 µL of the S^*^ strand (10 µM) was added, and the mixture was incubated at room temperature for over 2 h to ensure complete ligation. After ligation, no additional purification step was performed to remove the splint strand S^*^. Prior to HPLC analysis, the reaction volume was adjusted to 120 µL by adding 100 µL of 0.1 M TEAA aqueous solution.

### 4.8 Repeated Decoding and Re‐Encoding

FAM‐labeled DNA tags were mixed according to rules to encode the target message (300 nM for each tag). The encoded samples were then decoded using a standard HPLC method (injection volume, 100 µL). For the repeated‐decoding experiment, after each HPLC run the eluate was collected and purified using 3 kDa ultra centrifugal filter to remove salts. The purified sample was dried using a vacuum centrifugal concentrator, reconstituted in mobile phase A, and reinjected for the next decoding cycle. For the re‐encoding experiment, during HPLC decoding, fractions corresponding to each DNA tag peak were collected individually and purified/concentrated as described above. The recovered DNA tags were then reassembled according to a new target message to generate a re‐encoded sample, which was then subjected to HPLC readout again.

## Author Contributions

D.H., L.H., and L.C. conceived the study. Under the guidance of D.H. and L.H., L.C. designed and performed the main experiments, analysed the data, and drafted the manuscript. H.W. contributed to methodology development and experimental investigation. H.G. contributed to validation and formal analysis. S.C., Z.L., and Z.H. contributed to formal analysis. L.H. contributed to project administration and manuscript preparation. D.H. supervised the study and revised the manuscript. All authors discussed the results and approved the final manuscript.

## Conflicts of Interest

The authors declare no conflicts of interest.

## Supporting information




**Supporting File**: advs76492‐sup‐0001‐SuppMat.docx.

## Data Availability

The data that support the findings of this study are available in the Supporting Information of this article.
