# Supplementary material for: Time Resolved DNA Barcodes for Information Encoding and Dynamic Encryption
Source: Adv Sci (Weinh). 2026 Jul 9:e76492. Online ahead of print. doi: 10.1002/advs.76492 (PMC13348338; doi:10.1002/advs.76492)
Supplement: Supplementary file 1 — Supporting File: advs76492‐sup‐0001‐SuppMat.docx. [file ADVS-9999-e76492-s001.docx]

**Supplementary Information**

Time-resolved DNA barcodes for information encoding and dynamic encryption

Likang Chu,^[a], [b], [c], #^ Haixia Wang,^[a], #^ Haiyan Gao,^[a], [c]^ Sixian Chen,^[a], [d]^ Zilong Li,^[a], [e]^ Zhanyihao Hao,^[a], [c]^ Lei He,^[a],^ * Da Han^[a],^ ^[f],^ *

[a] The Key Laboratory of Zhejiang Province for Aptamers and Theranostics, Hangzhou Institute of Medicine (HIM), Chinese Academy of Sciences, Hangzhou, Zhejiang, China

[b] University of Chinese Academy of Sciences, Beijing, China

[c] School of Molecular Medicine, Hangzhou Institute for Advanced Study, University of Chinese Academy of Sciences, Hangzhou, Zhejiang, China

[d] College of Materials Science and Engineering, Zhejiang University of Technology, Hangzhou, Zhejiang, China

[e] College of Pharmaceutical Science, Zhejiang University of Technology, Hangzhou, Zhejiang, China

[f] Institute of Molecular Medicine (IMM), Renji Hospital, School of Medicine, Shanghai Jiao Tong University, Shanghai, China

^#^Likang Chu and Haixia Wang contributed equally to this work

*Correspondence: Da Han, dahan@sjtu.edu.cn; Lei He, helei@hnu.edu.cn

**Abbreviations**

| **Abbreviation** | **Full term** |
| --- | --- |
| DTB / DTBs | DNA Temporal Barcode / DNA Temporal Barcodes |
| SDT | Split DNA tag |
| ssDNA | Single-stranded DNA |
| HPLC | High-Performance Liquid Chromatography |
| P | Phosphate |
| Acr | Acrydite |
| Mb | Methylene blue |
| Fer | Ferrocene |
| Dig | Digoxigenin |
| Pyr | Pyrene |
| Bio | Biotin |
| AF647 | Alexa Fluor 647 |

**Supplementary Figures**


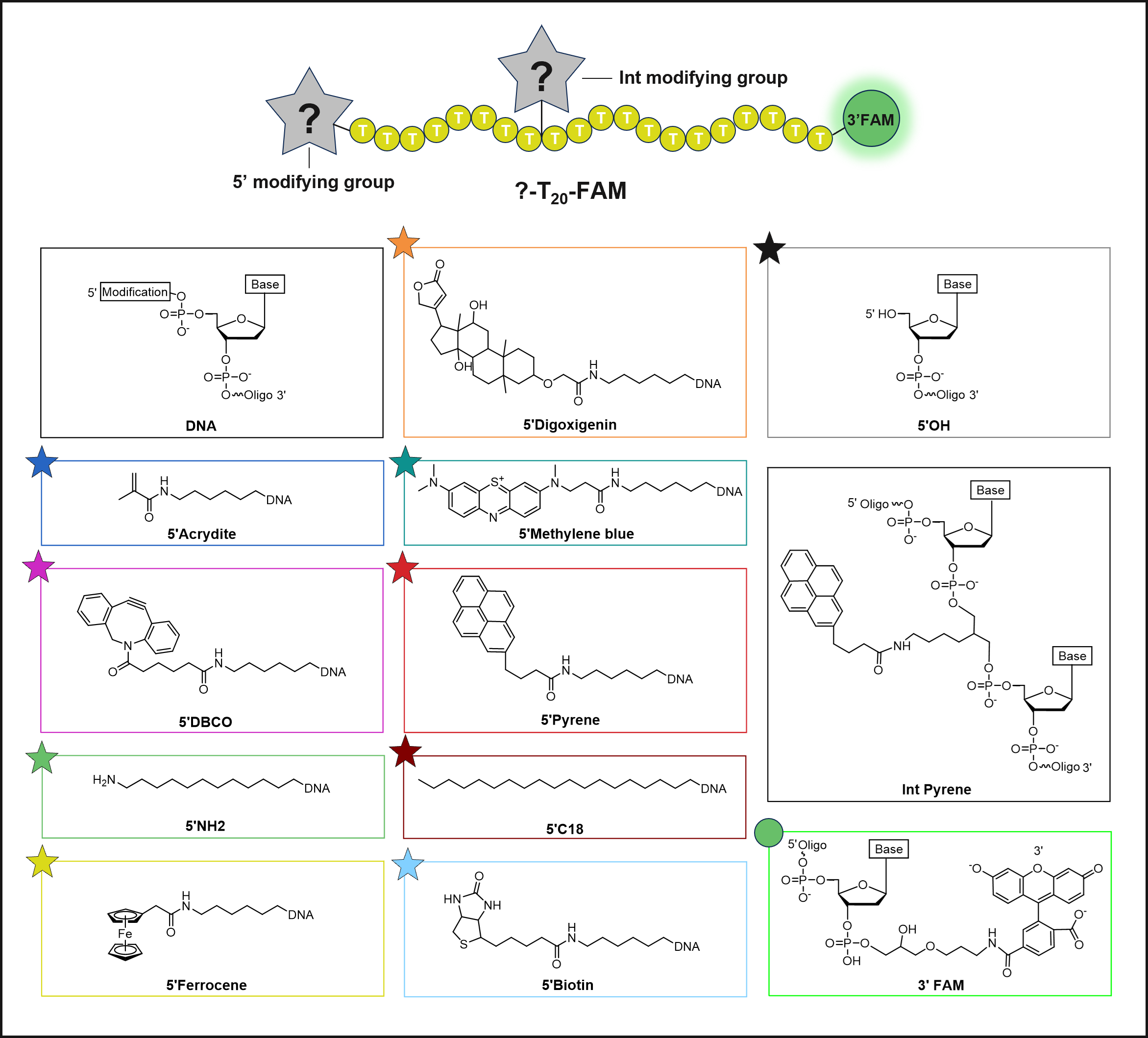


**Figure S1.** Molecular structures of modification groups on DNA tags. 5′ indicates modification at the 5′ end of the ssDNA, 3′ indicates modification at the 3′ end of the ssDNA, and Int indicates internal modification within the ssDNA.

**
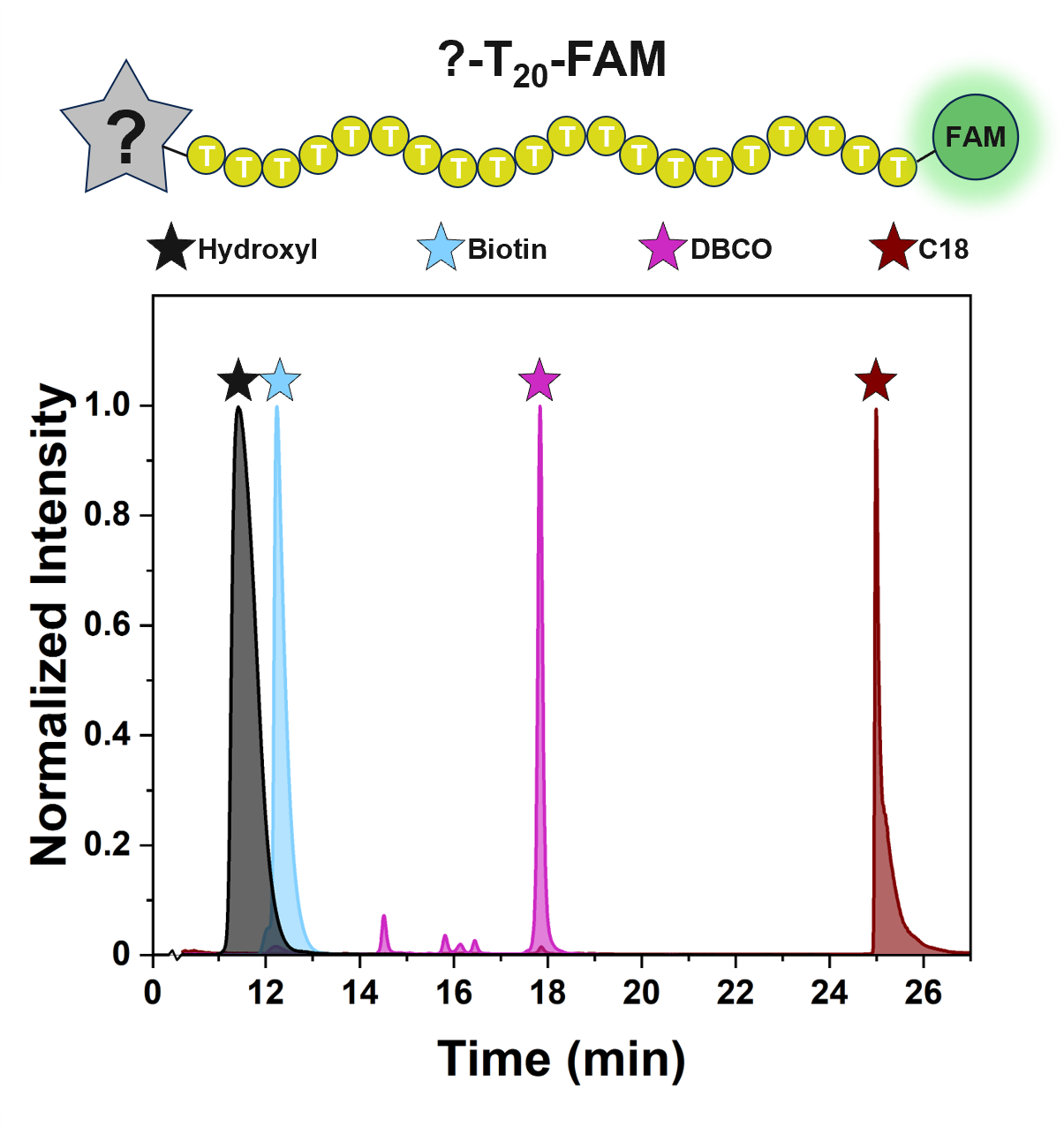
**

**Figure S2.** Effect of 5′ modification types on the retention behavior of DNA tags in HPLC. Shown are the HPLC signal peaks of T_20_-FAM modified at the 5′ end with hydroxyl (OH), biotin (Bio), dibenzocyclooctyne (DBCO), and octadecyl (C18), respectively. The corresponding retention times were 11.41 min for OH-T_20_-FAM, 12.23 min for Bio-T_20_-FAM, 17.83 min for DBCO-T_20_-FAM, and 24.99 min for C18-T_20_-FAM. The intensities of fluorescence signal peaks were normalized.

**
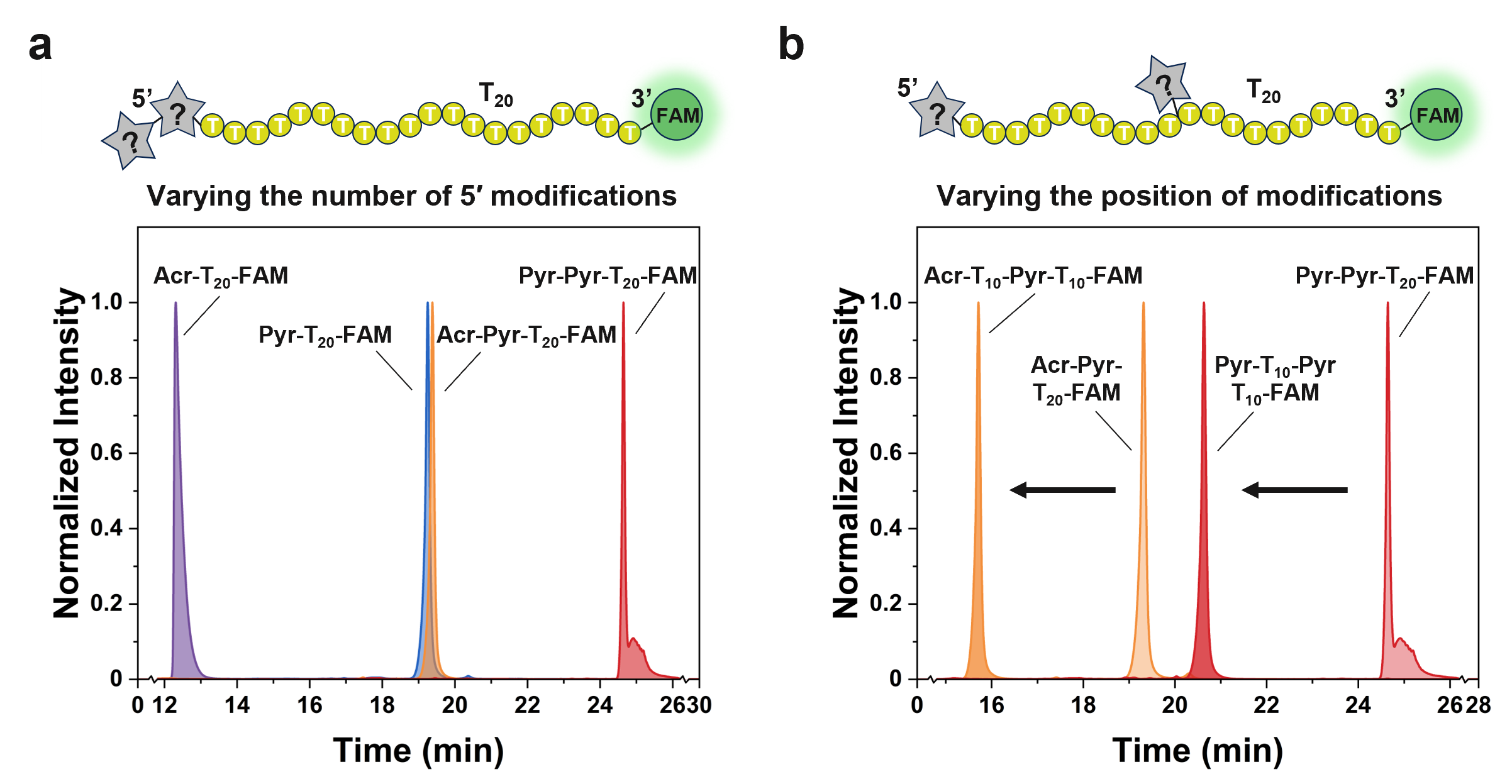
**

**Figure S3.** Effect of the number and position of modifications on the retention behavior of DNA tags in HPLC. (a) Effect of the number of 5′ modifications. When an additional pyrene (Pyr) was introduced at the 5′, the retention times of Acr-T_20_-FAM (12.32 min) and Pyr-T_20_-FAM (19.26 min) increased to 19.38 min for Acr-Pyr-T_20_-FAM and 24.65 min for Pyr-Pyr-T_20_-FAM, respectively. (b) Effect of the modification position. When the modification was relocated to the middle of the DNA tags, the retention times decreased significantly: the retention time of Acr-Pyr-T_20_-FAM shortened from 19.32 min to 15.71 min for Acr-T_10_-Pyr-T_10_-FAM, and that of Pyr-Pyr-T_20_-FAM shortened from 24.65 min to 20.64 min for Pyr-T_10_-Pyr-T_10_-FAM. The intensities of fluorescence signal peaks were normalized.

**
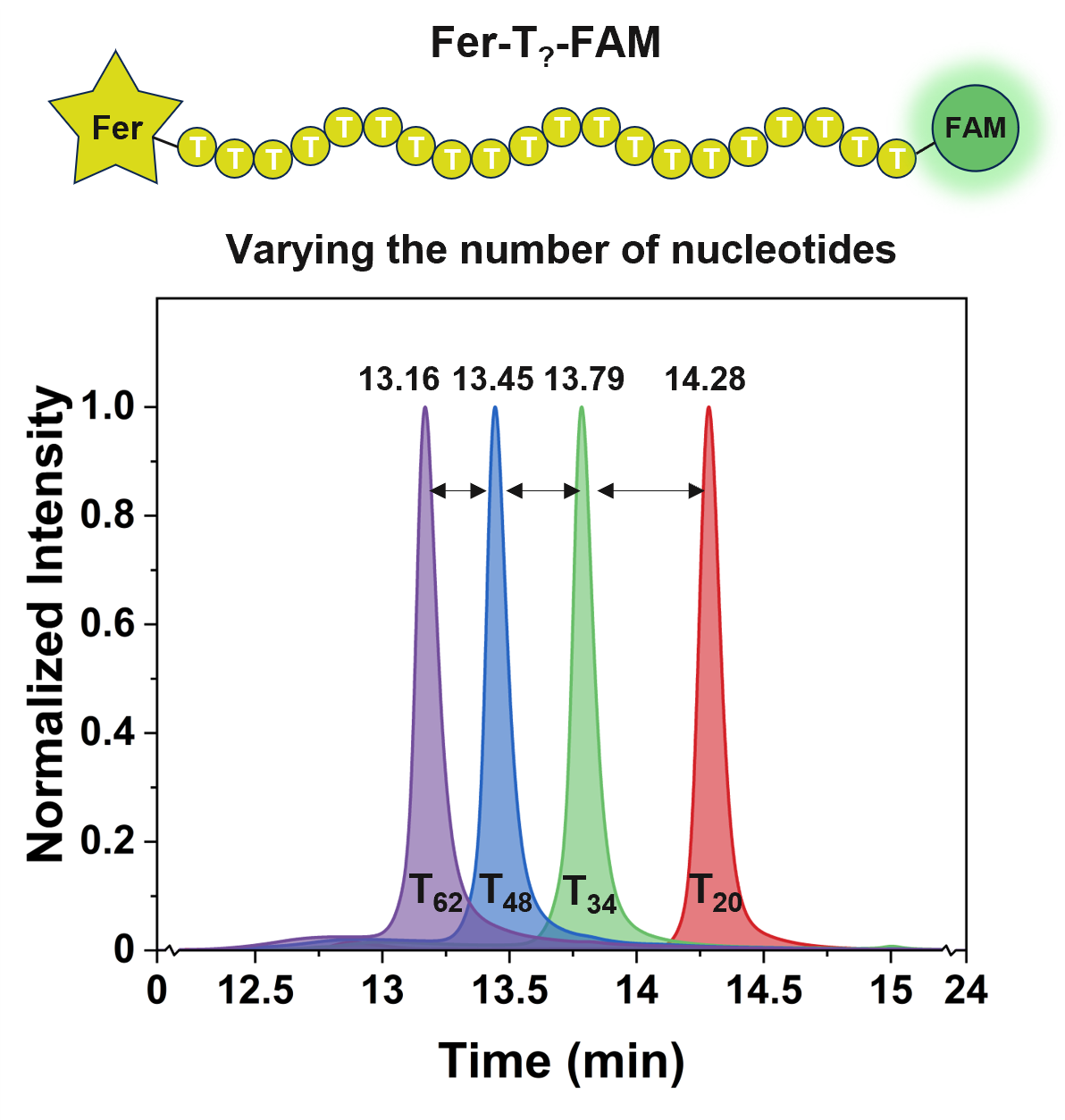
**

**Figure S4.** Impact of sequence length on the retention time of DNA tags. The DNA tags used in this experiment had a ferrocene (Fer) group at the 5′ end, and their sequences were designed with varying numbers of poly T. HPLC chromatograms were obtained for DNA tags with different numbers of T. The retention times increased sequentially as follows: Fer-T_62_-FAM (13.16 min), Fer-T_48_-FAM (13.45 min), Fer-T_34_-FAM (13.79 min), and Fer-T_20_-FAM (14.28 min). The intensities of fluorescence signal peaks were normalized.

**
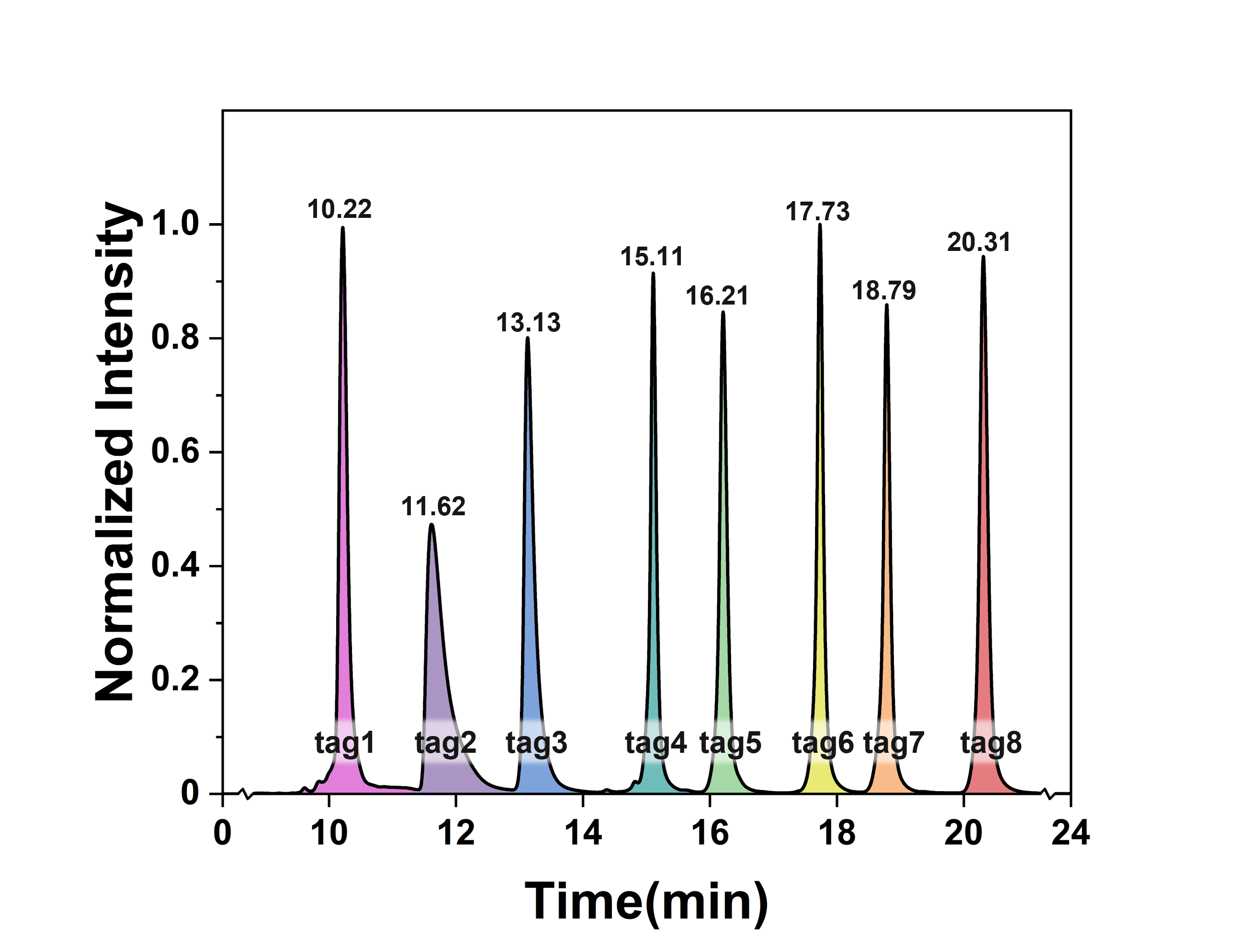
**

**Figure S5.** Orthogonality testing of DNA Temporal Barcodes for information encoding. Chromatogram obtained from simultaneous HPLC analysis of a mixture of FAM-labeled DNA tag1 to tag8, showing that the retention times of all DNA tags were consistent with those measured individually. The concentration of each DNA tag was 100 nmol/L. The intensities of fluorescence signal peaks were normalized.

**
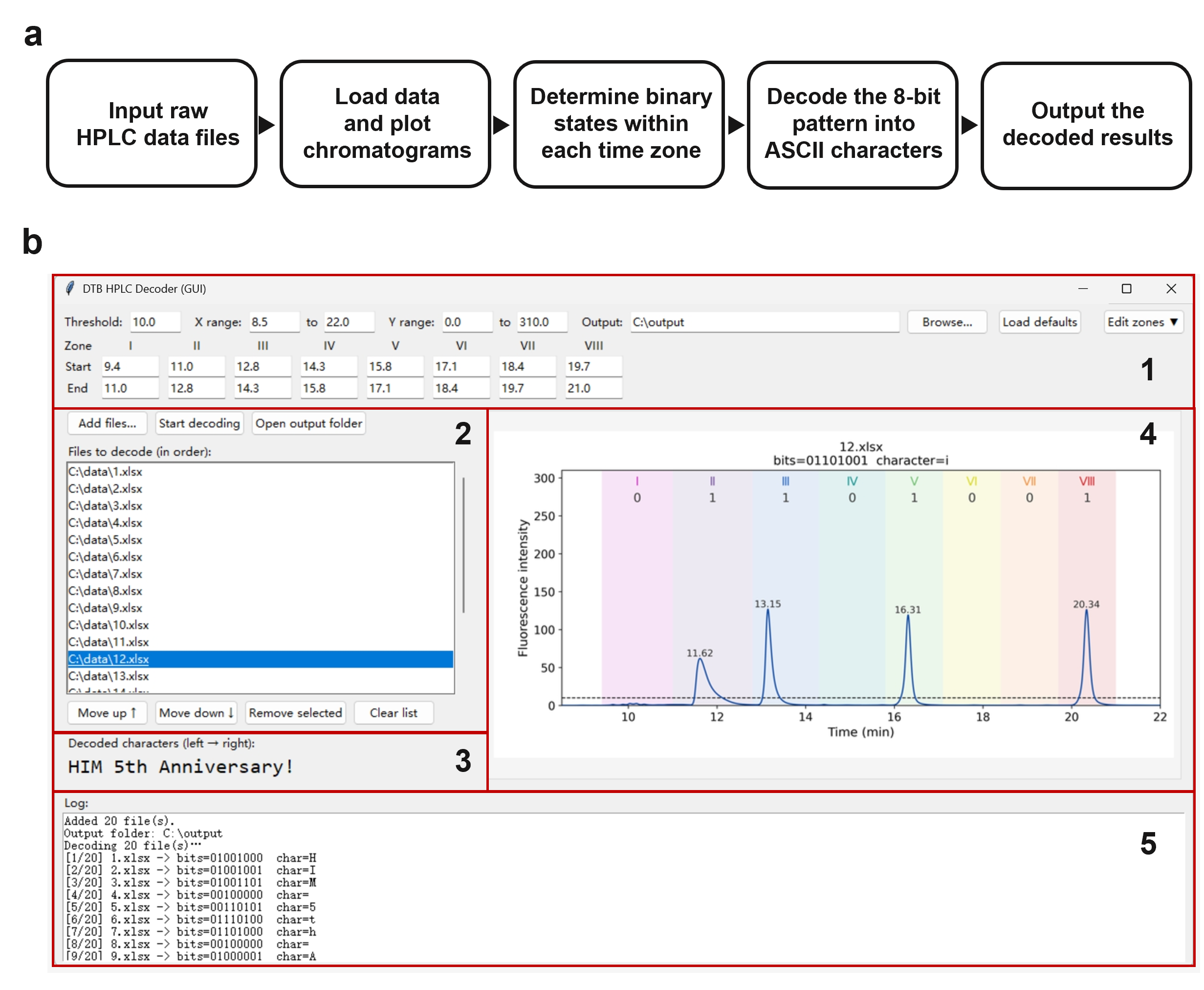
**

**Figure S6.** Graphical user interface of the DTB HPLC decoder. (a) Schematic workflow of the DTB HPLC decoder. (b) Graphical user interface of the DTB HPLC decoder: (1) Panel for setting decoding parameters, including fluorescence threshold, x– and y–axis ranges, and boundaries of the eight temporal zones. A one-click “default” option loads the parameter set optimized for the DNA tags and HPLC conditions used in this study, while all parameters remain adjustable when different tags or HPLC methods are applied. (2) File management panel for importing raw HPLC chromatogram data and arranging them in the desired decoding order before starting the analysis. (3) Output panel displaying the decoded characters from left to right for all imported samples. (4) Preview window showing the decoding chromatogram for the currently selected sample; high-resolution chromatograms are simultaneously exported to the output folder. (5) Log window reporting program status and a real-time record of the decoding process. All code was written in Python.

**
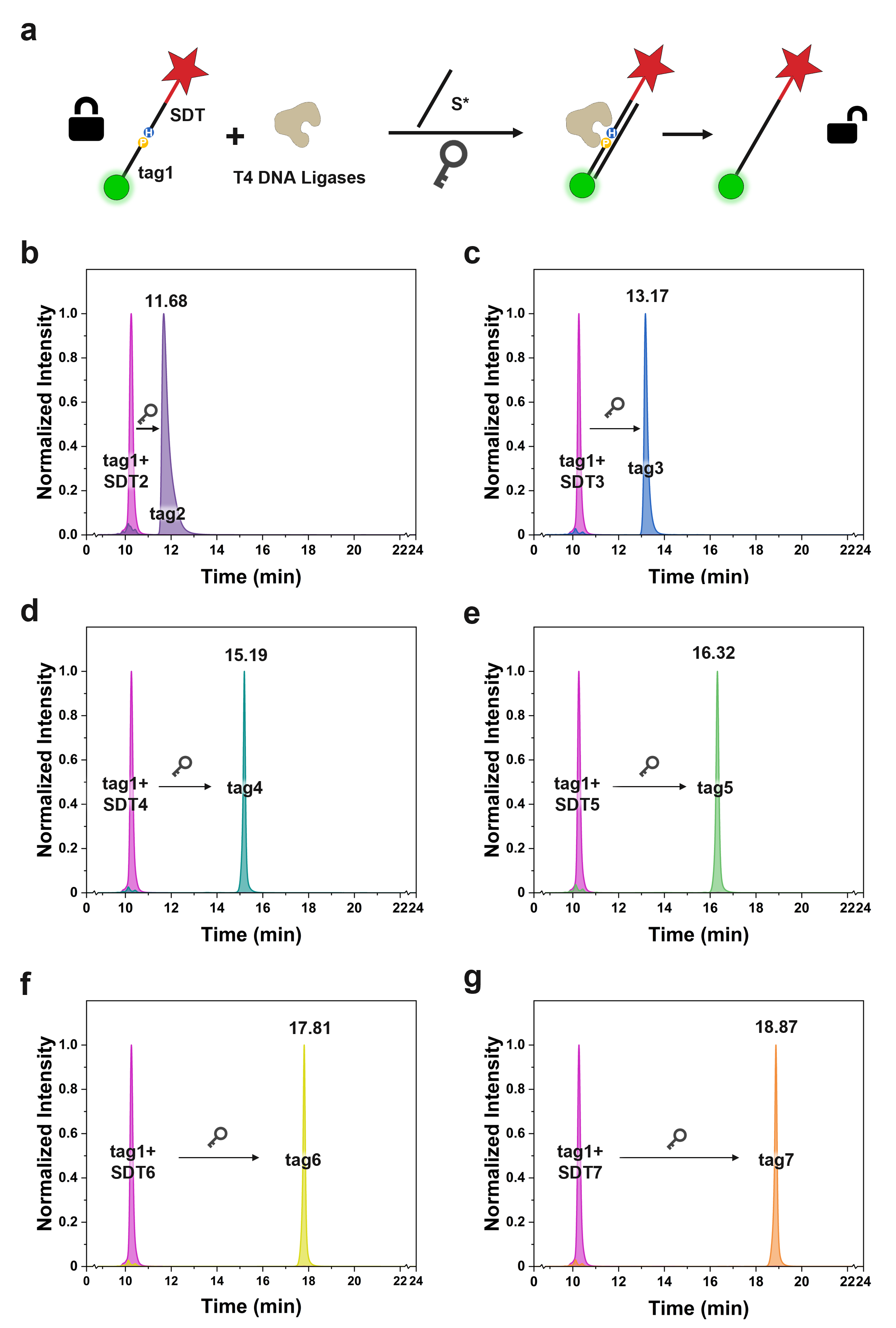
**

**Figure S7.** Feasibility validation of the dynamic encryption mechanism. (a) Schematic illustration of the information encryption principle using DNA tag1 and Split DNA tag (SDT). (b–g) Key-mediated DNA ligation reactions were performed for SDT2 to SDT7 in the presence of tag1 and T4 DNA ligase. HPLC analyses were conducted before and after the reactions to characterize the encrypted and decrypted states of the information. The intensities of fluorescence signal peaks were normalized.


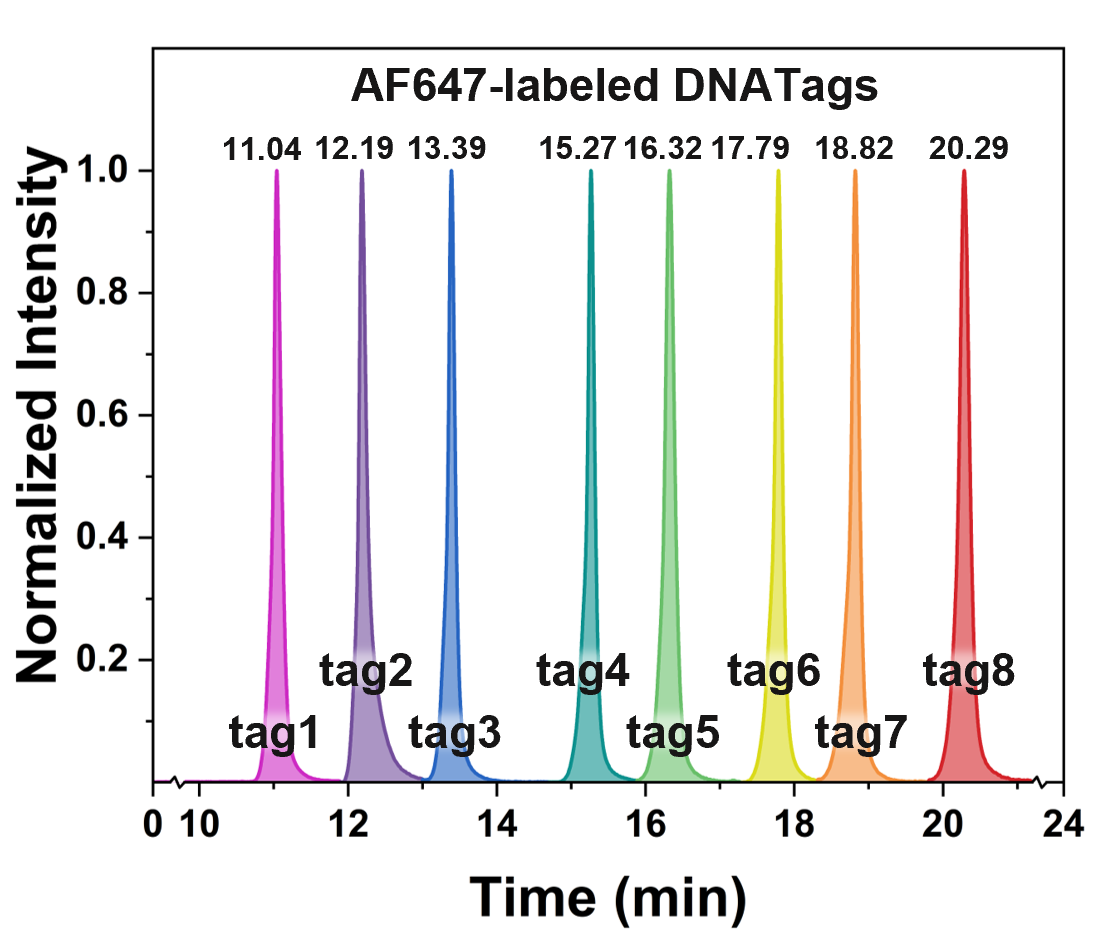


**Figure S8.** HPLC chromatograms of eight AF647-labeled DNA tags. The retention times of the AF647-labeled tags closely matched those of their FAM-labeled counterparts in a one-to-one manner. The AF647-tag set was designed using the same strategy as that for the FAM-labeled DNA tags, and the corresponding sequences are listed in Table S8. The x-axis represents retention time, and the y-axis represents normalized AF647 fluorescence intensity.


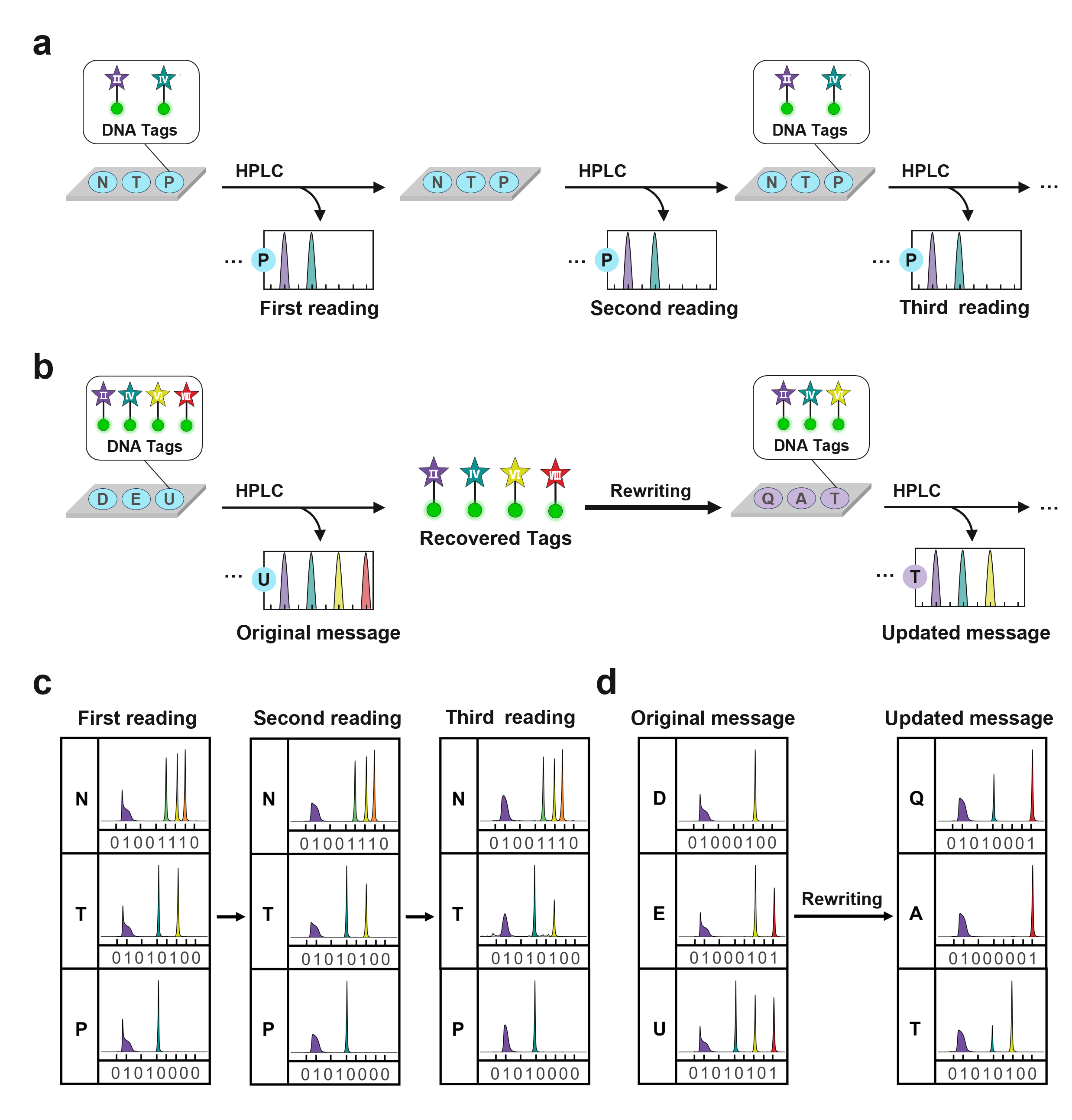


**Figure S9.** Repeated readout and rewriting of information. (a) Schematic illustration of repeated readout. DNA tags encoding the message “NTP” were injected into the HPLC system for decoding. The tags recovered after each decoding run were reinjected into the HPLC system for subsequent cycles, enabling multi-round readout. (b) Schematic illustration of information rewriting. DNA tags encoding the message “DEU” were injected into the HPLC system for decoding. Simultaneously, leveraging the high separation efficiency and peak collection capability of the HPLC, each DNA tag was sequentially recovered. The recovered tags were then reassembled to encode a new message, “QAT,” thereby achieving information rewriting. (c) HPLC decoding results over three consecutive readout cycles. (d) HPLC decoding results before and after rewriting. FAM-labeled DNA tags were used throughout. The y-axis represents normalized FAM fluorescence intensity, and the x-axis represents retention time (8–22 min).

**Supplementary Tables**

**Table S1.** Retention-time comparison of DNA Temporal Barcodes analyzed individually and as a mixture.

|  | tag1  (min) | tag2  (min) | tag3  (min) | tag4  (min) | tag5  (min) | tag6  (min) | tag7  (min) | tag8  (min) |
| --- | --- | --- | --- | --- | --- | --- | --- | --- |
| Individual injection | 10.306 | 11.536 | 13.200 | 15.172 | 16.289 | 17.765 | 18.830 | 20.320 |
| Mixed injection | 10.217 | 11.615 | 13.128 | 15.107 | 16.208 | 17.734 | 18.785 | 20.307 |
| Absolute difference | 0.089 | 0.079 | 0.072 | 0.065 | 0.081 | 0.031 | 0.045 | 0.013 |

Note: Retention times of each DNA tag measured under two analytical modes are summarized in this table. 'Individual injection' refers to HPLC analysis of each FAM-labeled DNA tag separately. 'Mixed injection' refers to simultaneous HPLC analysis of a mixture containing all eight FAM-labeled DNA tags. 'Absolute difference' was calculated as the absolute value of the retention-time difference between the two analytical modes for each DNA tag.

**Table S2.** Short-term repeatability analysis of DNA Temporal Barcodes retention time.

|  | tag1  (min) | tag2  (min) | tag3  (min) | tag4  (min) | tag5  (min) | tag6  (min) | tag7  (min) | tag8  (min) |
| --- | --- | --- | --- | --- | --- | --- | --- | --- |
| Test 1 | 10.218 | 11.597 | 13.093 | 15.124 | 16.259 | 17.729 | 18.798 | 20.276 |
| Test 2 | 10.218 | 11.596 | 13.093 | 15.128 | 16.264 | 17.739 | 18.808 | 20.286 |
| Test 3 | 10.225 | 11.598 | 13.102 | 15.128 | 16.263 | 17.740 | 18.813 | 20.285 |
| Test 4 | 10.223 | 11.591 | 13.096 | 15.132 | 16.265 | 17.737 | 18.807 | 20.283 |
| Test 5 | 10.222 | 11.592 | 13.092 | 15.122 | 16.25 | 17.728 | 18.803 | 20.283 |
| Test 6 | 10.226 | 11.595 | 13.106 | 15.134 | 16.271 | 17.748 | 18.821 | 20.293 |
| Test 7 | 10.217 | 11.583 | 13.096 | 15.129 | 16.262 | 17.740 | 18.806 | 20.275 |
| Test 8 | 10.218 | 11.578 | 13.104 | 15.129 | 16.263 | 17.741 | 18.807 | 20.279 |
| Test 9 | 10.220 | 11.579 | 13.102 | 15.130 | 16.264 | 17.744 | 18.812 | 20.286 |
| Test 10 | 10.222 | 11.595 | 13.097 | 15.137 | 16.268 | 17.744 | 18.815 | 20.286 |
| CV (%) | 0.031% | 0.065% | 0.038% | 0.029% | 0.034% | 0.036% | 0.035% | 0.026% |

Note: The data in the table represent the retention times of DNA Temporal Barcodes measured repeatedly over a short period. 'Test 1' to 'Test 10' refer to ten consecutive runs, and 'CV' denotes the coefficient of variation of the retention time for each DNA tag.

**Table S3.** Long-term stability assessment of DNA Temporal Barcodes retention time.

|  | tag1  (min) | tag2  (min) | tag3  (min) | tag4  (min) | tag5  (min) | tag6  (min) | tag7  (min) | tag8  (min) |
| --- | --- | --- | --- | --- | --- | --- | --- | --- |
| Day 1 | 10.217 | 11.615 | 13.128 | 15.107 | 16.208 | 17.734 | 18.785 | 20.307 |
| Day 40 | 10.226 | 11.595 | 13.106 | 15.134 | 16.271 | 17.748 | 18.821 | 20.293 |
| Day 47 | 10.247 | 11.632 | 13.153 | 15.198 | 16.326 | 17.826 | 18.893 | 20.392 |
| CV (%) | 0.150% | 0.159% | 0.179% | 0.309% | 0.363% | 0.279% | 0.292% | 0.264% |

Note: DNA tag1–8 were stored as 20 μM stock solutions in DEPC-treated water at 4 °C. Before analysis, equal volumes of the DNA tag1–8 stocks were mixed and diluted with mobile phase A to 100 nM for each DNA tag. HPLC analyses were performed on Day 1, Day 40, and Day 47. 'CV' denotes the coefficient of variation of retention time across the three measurements.

**Table S4.** Characters, ASCII binary codes, and the associated DNA Temporal Barcode mixtures used for information encoding. (Standard ASCII code table).

| **Index** | **characters** | **Binary codes** | **DTB**  **mixtures** | **Index** | **characters** | **Binary codes** | **DTB**  **mixtures** | |
| --- | --- | --- | --- | --- | --- | --- | --- | --- |
| 0 | NUL | 00000000 | – | 36 | $ | 00100100 | III VI | |
| 1 | SOH | 00000001 | VII | 37 | % | 00100101 | Ⅲ Ⅵ Ⅷ | |
| 2 | STX | 00000010 | VII | 38 | & | 00100110 | Ⅲ Ⅵ Ⅶ | |
| 3 | ETX | 00000011 | VII VIII | 39 | ' | 00100111 | Ⅲ Ⅵ Ⅶ Ⅷ | |
| 4 | EOT | 00000100 | VI | 40 | ( | 00101000 | Ⅲ Ⅴ | |
| 5 | ENQ | 00000101 | VI VIII | 41 | ) | 00101001 | Ⅲ Ⅴ Ⅷ | |
| 6 | ACK | 00000110 | VI VII | 42 | * | 00101010 | Ⅲ Ⅴ Ⅶ | |
| 7 | BEL | 00000111 | VI VII VIII | 43 | + | 00101011 | Ⅲ Ⅴ Ⅶ Ⅷ | |
| 8 | BS | 00001000 | V | 44 | , | 00101100 | Ⅲ Ⅴ Ⅵ | |
| 9 | HT | 00001001 | V VIII | 45 | - | 00101101 | Ⅲ Ⅴ Ⅵ Ⅷ | |
| 10 | LF | 00001010 | V VII | 46 | . | 00101110 | Ⅲ Ⅴ Ⅵ Ⅶ | |
| 11 | VT | 00001011 | V VII VIII | 47 | / | 00101111 | Ⅲ Ⅴ Ⅵ Ⅶ Ⅷ | |
| 12 | FF | 00001100 | V VI | 48 | 0 | 00110000 | Ⅲ Ⅳ | |
| 13 | CR | 00001101 | V VI VIII | 49 | 1 | 00110001 | Ⅲ Ⅳ Ⅷ | |
| 14 | SO | 00001110 | V VI VII | 50 | 2 | 00110010 | Ⅲ Ⅳ Ⅶ | |
| 15 | SI | 00001111 | V VI VII VIII | 51 | 3 | 00110011 | Ⅲ Ⅳ Ⅶ Ⅷ | |
| 16 | DLE | 00010000 | IV | 52 | 4 | 00110100 | Ⅲ Ⅳ Ⅵ | |
| 17 | DC1 | 00010001 | IV VIII | **53** | **5** | **00110101** | **Ⅲ Ⅳ Ⅵ Ⅷ** | |
| 18 | DC2 | 00010010 | IV VII | 54 | 6 | 00110110 | Ⅲ Ⅳ Ⅵ Ⅶ | |
| 19 | DC3 | 00010011 | IV VII VIII | 55 | 7 | 00110111 | Ⅲ Ⅳ Ⅵ Ⅶ Ⅷ | |
| 20 | DC4 | 00010100 | IV VI | 56 | 8 | 00111000 | Ⅲ Ⅳ Ⅴ | |
| 21 | NAK | 00010101 | IV VI VIII | 57 | 9 | 00111001 | Ⅲ Ⅳ Ⅴ Ⅷ | |
| 22 | SYN | 00010110 | IV VI VII | 58 | : | 00111010 | Ⅲ Ⅳ Ⅴ Ⅶ | |
| 23 | ETB | 00010111 | IV VI VII VIII | 59 | ; | 00111011 | Ⅲ Ⅳ Ⅴ Ⅶ Ⅷ | |
| 24 | CAN | 00011000 | IV V | 60 | < | 00111100 | Ⅲ Ⅳ Ⅴ Ⅵ | |
| 25 | EM | 00011001 | IV V VIII | 61 | = | 00111101 | Ⅲ Ⅳ Ⅴ Ⅵ Ⅷ | |
| 26 | SUB | 00011010 | IV V VII | 62 | > | 00111110 | Ⅲ Ⅳ Ⅴ Ⅵ Ⅶ | |
| 27 | ESC | 00011011 | IV V VII VIII | 63 | ? | 00111111 | Ⅲ Ⅳ Ⅴ Ⅵ Ⅶ Ⅷ | |
| 28 | FS | 00011100 | IV V VI | 64 | @ | 01000000 | Ⅱ | |
| 29 | GS | 00011101 | IV V VI VIII | **65** | **A** | **01000001** | **Ⅱ Ⅷ** | |
| 30 | RS | 00011110 | IV V VI VII | 66 | B | 01000010 | Ⅱ Ⅶ | |
| 31 | US | 00011111 | IV V VI VII VIII | 67 | C | 01000011 | Ⅱ Ⅶ Ⅷ | |
| **32** | **Spcae** | **00100000** | **III** | **68** | **D** | **01000100** | **Ⅱ Ⅵ** | |
| **33** | **!** | **00100001** | **III VIII** | **69** | **E** | **01000101** | **Ⅱ Ⅵ Ⅷ** | |
| 34 | " | 00100010 | III VII | 70 | F | 01000110 | Ⅱ Ⅵ Ⅶ | |
| 35 | # | 00100011 | III VII VIII | 71 | G | 01000111 | Ⅱ Ⅵ Ⅶ Ⅷ | |
| **72** | **H** | **01001000** | **Ⅱ Ⅴ** | **108** | **l** | **01101100** | **Ⅱ Ⅲ Ⅴ Ⅵ** | |
| **73** | **I** | **01001001** | **Ⅱ Ⅴ Ⅷ** | 109 | m | 01101101 | Ⅱ Ⅲ Ⅴ Ⅵ Ⅷ | |
| 74 | J | 01001010 | Ⅱ Ⅴ Ⅶ | **110** | **n** | **01101110** | **Ⅱ Ⅲ Ⅴ Ⅵ Ⅶ** | |
| 75 | K | 01001011 | Ⅱ Ⅴ Ⅶ Ⅷ | 111 | o | 01101111 | Ⅱ Ⅲ Ⅴ Ⅵ Ⅶ Ⅷ | |
| 76 | L | 01001100 | Ⅱ Ⅴ Ⅵ | 112 | p | 01110000 | Ⅱ Ⅲ Ⅳ | |
| **77** | **M** | **01001101** | **Ⅱ Ⅴ Ⅵ Ⅷ** | 113 | q | 01110001 | Ⅱ Ⅲ Ⅳ Ⅷ | |
| **78** | **N** | **01001110** | **Ⅱ Ⅴ Ⅵ Ⅶ** | **114** | **r** | **01110010** | **Ⅱ Ⅲ Ⅳ Ⅶ** | |
| 79 | O | 01001111 | Ⅱ Ⅴ Ⅵ Ⅶ Ⅷ | **115** | **s** | **01110011** | **Ⅱ Ⅲ Ⅳ Ⅶ Ⅷ** | |
| **80** | **P** | **01010000** | **Ⅱ Ⅳ** | **116** | **t** | **01110100** | **Ⅱ Ⅲ Ⅳ Ⅵ** | |
| **81** | **Q** | **01010001** | **Ⅱ Ⅳ Ⅷ** | 117 | u | 01110101 | Ⅱ Ⅲ Ⅳ Ⅵ Ⅷ | |
| 82 | R | 01010010 | Ⅱ Ⅳ Ⅶ | **118** | **v** | **01110110** | **Ⅱ Ⅲ Ⅳ Ⅵ Ⅶ** | |
| 83 | S | 01010011 | Ⅱ Ⅳ Ⅶ Ⅷ | 119 | w | 01110111 | Ⅱ Ⅲ Ⅳ Ⅵ Ⅶ Ⅷ | |
| **84** | **T** | **01010100** | **Ⅱ Ⅳ Ⅵ** | 120 | x | 01111000 | Ⅱ Ⅲ Ⅳ Ⅴ | |
| **85** | **U** | **01010101** | **Ⅱ Ⅳ Ⅵ Ⅷ** | **121** | **y** | **01111001** | **Ⅱ Ⅲ Ⅳ Ⅴ Ⅷ** | |
| 86 | V | 01010110 | Ⅱ Ⅳ Ⅵ Ⅶ | 122 | z | 01111010 | Ⅱ Ⅲ Ⅳ Ⅴ Ⅶ | |
| 87 | W | 01010111 | Ⅱ Ⅳ Ⅵ Ⅶ Ⅷ | 123 | { | 01111011 | Ⅱ Ⅲ Ⅳ Ⅴ Ⅶ Ⅷ | |
| 88 | X | 01011000 | Ⅱ Ⅳ Ⅴ | 124 | \| | 01111100 | Ⅱ Ⅲ Ⅳ Ⅴ Ⅵ | |
| 89 | Y | 01011001 | Ⅱ Ⅳ Ⅴ Ⅷ | 125 | } | 01111101 | Ⅱ Ⅲ Ⅳ Ⅴ Ⅵ Ⅷ | |
| 90 | Z | 01011010 | Ⅱ Ⅳ Ⅴ Ⅶ | 126 | ~ | 01111110 | Ⅱ Ⅲ Ⅳ Ⅴ Ⅵ Ⅶ | |
| 91 | [ | 01011011 | Ⅱ Ⅳ Ⅴ Ⅶ Ⅷ | 127 | DEL | 01111111 | Ⅱ Ⅲ Ⅳ Ⅴ Ⅵ Ⅶ Ⅷ | |
| 92 | \ | 01011100 | Ⅱ Ⅳ Ⅴ Ⅵ |  | | | |  |
| 93 | ] | 01011101 | Ⅱ Ⅳ Ⅴ Ⅵ Ⅷ |  |  |  |  |  |
| 94 | ^ | 01011110 | Ⅱ Ⅳ Ⅴ Ⅵ Ⅶ |  |  |  |  |  |
| 95 | _ | 01011111 | Ⅱ Ⅳ Ⅴ Ⅵ Ⅶ Ⅷ |  |  |  |  |  |
| 96 | ` | 01100000 | Ⅱ Ⅲ |  |  |  |  |  |
| **97** | **a** | **01100001** | **Ⅱ Ⅲ Ⅷ** |  |  |  |  |  |
| 98 | b | 01100010 | Ⅱ Ⅲ Ⅶ |  |  |  |  | |
| **99** | **c** | **01100011** | **Ⅱ Ⅲ Ⅶ Ⅷ** |  |  |  |  | |
| 100 | d | 01100100 | Ⅱ Ⅲ Ⅵ |  |  |  |  | |
| **101** | **e** | **01100101** | **Ⅱ Ⅲ Ⅵ Ⅷ** |  |  |  |  | |
| 102 | f | 01100110 | Ⅱ Ⅲ Ⅵ Ⅶ |  |  |  |  | |
| 103 | g | 01100111 | Ⅱ Ⅲ Ⅵ Ⅶ Ⅷ |  |  |  |  | |
| **104** | **h** | **01101000** | **Ⅱ Ⅲ Ⅴ** |  |  |  |  | |
| **105** | **i** | **01101001** | **Ⅱ Ⅲ Ⅴ Ⅷ** |  |  |  |  | |
| 106 | j | 01101010 | Ⅱ Ⅲ Ⅴ Ⅶ |  |  |  |  | |
| 107 | k | 01101011 | Ⅱ Ⅲ Ⅴ Ⅶ Ⅷ |  |  |  |  | |

**Note:** Bolded entries in the table indicate the characters involved in this study. Ⅰ, Ⅱ, Ⅲ, Ⅳ, Ⅴ, Ⅵ, Ⅶ, and Ⅷ correspond to tag1, tag2, tag3, tag4, tag5, tag6, tag7, and tag8, respectively.

**Table S5.** The characters, their corresponding ASCII binary codes, and the associated DNA Temporal Barcode mixtures used for information encoding. (Extended ASCII code table).

| **Index** | **characters** | **Binary codes** | **DTB**  **mixtures** | **Index** | **characters** | **Binary codes** | **DTB**  **mixtures** |
| --- | --- | --- | --- | --- | --- | --- | --- |
| 128 | € | 10000000 | Ⅰ | 163 | £ | 10100011 | Ⅰ Ⅲ Ⅶ Ⅷ |
| 129 | undefined | 10000001 | Ⅰ Ⅷ | 164 | ¤ | 10100100 | Ⅰ Ⅲ Ⅵ |
| 130 | ‚ | 10000010 | Ⅰ Ⅶ | 165 | ¥ | 10100101 | Ⅰ Ⅲ Ⅵ Ⅷ |
| 131 | ƒ | 10000011 | Ⅰ Ⅶ Ⅷ | 166 | ¦ | 10100110 | Ⅰ Ⅲ Ⅵ Ⅶ |
| 132 | „ | 10000100 | Ⅰ Ⅵ | 167 | § | 10100111 | Ⅰ Ⅲ Ⅵ Ⅶ Ⅷ |
| 133 | … | 10000101 | Ⅰ Ⅵ Ⅷ | 168 | ¨ | 10101000 | Ⅰ Ⅲ Ⅴ |
| 134 | † | 10000110 | Ⅰ Ⅵ Ⅶ | 169 | © | 10101001 | Ⅰ Ⅲ Ⅴ Ⅷ |
| 135 | ‡ | 10000111 | Ⅰ Ⅵ Ⅶ Ⅷ | 170 | ª | 10101010 | Ⅰ Ⅲ Ⅴ Ⅶ |
| 136 | ˆ | 10001000 | ⅠⅤ | 171 | « | 10101011 | Ⅰ Ⅲ Ⅴ Ⅶ Ⅷ |
| 137 | ‰ | 10001001 | Ⅰ Ⅴ Ⅷ | 172 | ¬ | 10101100 | Ⅰ Ⅲ Ⅴ Ⅵ |
| 138 | Š | 10001010 | Ⅰ Ⅴ Ⅶ | 173 | ­ soft hyphen | 10101101 | Ⅰ Ⅲ Ⅴ Ⅵ Ⅷ |
| 139 | ‹ | 10001011 | Ⅰ Ⅴ Ⅶ Ⅷ | 174 | ® | 10101110 | Ⅰ Ⅲ Ⅴ Ⅵ Ⅶ |
| 140 | Œ | 10001100 | Ⅰ Ⅴ Ⅵ | 175 | ¯ | 10101111 | Ⅰ Ⅲ Ⅴ Ⅵ Ⅶ Ⅷ |
| 141 | undefined | 10001101 | Ⅰ Ⅴ Ⅵ Ⅷ | 176 | ° | 10110000 | Ⅰ Ⅲ Ⅳ |
| 142 | Ž | 10001110 | Ⅰ Ⅴ Ⅵ Ⅶ | 177 | ± | 10110001 | Ⅰ Ⅲ Ⅳ Ⅷ |
| 143 | undefined | 10001111 | ⅠⅤ Ⅵ Ⅶ Ⅷ | 178 | ² | 10110010 | Ⅰ Ⅲ Ⅳ Ⅶ |
| 144 | undefined | 10010000 | Ⅰ Ⅳ | 179 | ³ | 10110011 | Ⅰ Ⅲ Ⅳ Ⅶ Ⅷ |
| 145 | ‘ | 10010001 | Ⅰ Ⅳ Ⅷ | 180 | ´ | 10110100 | Ⅰ Ⅲ Ⅳ Ⅵ |
| 146 | ’ | 10010010 | Ⅰ Ⅳ Ⅶ | 181 | µ | 10110101 | Ⅰ Ⅲ Ⅳ Ⅵ Ⅷ |
| 147 | “ | 10010011 | Ⅰ Ⅳ Ⅶ Ⅷ | 182 | ¶ | 10110110 | Ⅰ Ⅲ Ⅳ Ⅵ Ⅶ |
| 148 | ” | 10010100 | Ⅰ Ⅳ Ⅵ | 183 | · | 10110111 | Ⅰ Ⅲ Ⅳ Ⅵ Ⅶ Ⅷ |
| 149 | • | 10010101 | Ⅰ Ⅳ Ⅵ Ⅷ | 184 | ¸ | 10111000 | Ⅰ Ⅲ Ⅳ Ⅴ |
| 150 | – | 10010110 | Ⅰ Ⅳ Ⅵ Ⅶ | 185 | ¹ | 10111001 | Ⅰ Ⅲ Ⅳ Ⅴ Ⅷ |
| 151 | — | 10010111 | Ⅰ Ⅳ Ⅵ Ⅶ Ⅷ | 186 | º | 10111010 | Ⅰ Ⅲ Ⅳ Ⅴ Ⅶ |
| 152 | ˜ | 10011000 | Ⅰ Ⅳ Ⅴ | 187 | » | 10111011 | Ⅰ Ⅲ Ⅳ Ⅴ Ⅶ Ⅷ |
| 153 | ™ | 10011001 | Ⅰ Ⅳ Ⅴ Ⅷ | 188 | ¼ | 10111100 | Ⅰ Ⅲ Ⅳ Ⅴ Ⅵ |
| 154 | š | 10011010 | Ⅰ Ⅳ Ⅴ Ⅶ | 189 | ½ | 10111101 | Ⅰ Ⅲ Ⅳ Ⅴ Ⅵ Ⅷ |
| 155 | › | 10011011 | Ⅰ Ⅳ Ⅴ Ⅶ Ⅷ | 190 | ¾ | 10111110 | Ⅰ Ⅲ Ⅳ Ⅴ Ⅵ Ⅶ |
| 156 | œ | 10011100 | Ⅰ Ⅳ Ⅴ Ⅵ | 191 | ¿ | 10111111 | Ⅰ Ⅲ Ⅳ Ⅴ Ⅵ Ⅶ Ⅷ |
| 157 | undefined | 10011101 | Ⅰ Ⅳ Ⅴ Ⅵ Ⅷ | 192 | À | 11000000 | ⅠⅡ |
| 158 | ž | 10011110 | Ⅰ Ⅳ Ⅴ Ⅵ Ⅶ | 193 | Á | 11000001 | Ⅰ Ⅱ Ⅷ |
| 159 | Ÿ | 10011111 | Ⅰ Ⅳ Ⅴ Ⅵ Ⅶ Ⅷ | 194 | Â | 11000010 | Ⅰ Ⅱ Ⅶ |
| 160 | NBSP | 10100000 | Ⅰ Ⅲ | 195 | Ã | 11000011 | Ⅰ Ⅱ Ⅶ Ⅷ |
| 161 | ¡ | 10100001 | Ⅰ Ⅲ Ⅷ | 196 | Ä | 11000100 | Ⅰ Ⅱ Ⅵ |
| 162 | ¢ | 10100010 | Ⅰ Ⅲ Ⅶ | 197 | Å | 11000101 | Ⅰ Ⅱ Ⅵ Ⅷ |
| 198 | Æ | 11000110 | Ⅰ Ⅱ Ⅵ Ⅶ | 234 | ê | 11101010 | Ⅰ Ⅱ Ⅲ Ⅴ Ⅶ |
| 199 | Ç | 11000111 | Ⅰ Ⅱ Ⅵ Ⅶ Ⅷ | 235 | ë | 11101011 | Ⅰ Ⅱ Ⅲ Ⅴ Ⅶ Ⅷ |
| 200 | È | 11001000 | Ⅰ Ⅱ Ⅴ | 236 | ì | 11101100 | Ⅰ Ⅱ Ⅲ Ⅴ Ⅵ |
| 201 | É | 11001001 | Ⅰ Ⅱ Ⅴ Ⅷ | 237 | í | 11101101 | Ⅰ Ⅱ Ⅲ Ⅴ Ⅵ Ⅷ |
| 202 | Ê | 11001010 | Ⅰ Ⅱ Ⅴ Ⅶ | 238 | î | 11101110 | Ⅰ Ⅱ Ⅲ Ⅴ Ⅵ Ⅶ |
| 203 | Ë | 11001011 | Ⅰ Ⅱ Ⅴ Ⅶ Ⅷ | 239 | ï | 11101111 | Ⅰ Ⅱ Ⅲ Ⅴ Ⅵ Ⅶ Ⅷ |
| 204 | Ì | 11001100 | Ⅰ Ⅱ Ⅴ Ⅵ | 240 | ð | 11110000 | Ⅰ Ⅱ Ⅲ Ⅳ |
| 205 | Í | 11001101 | Ⅰ Ⅱ Ⅴ Ⅵ Ⅷ | 241 | ñ | 11110001 | Ⅰ Ⅱ Ⅲ Ⅳ Ⅷ |
| 206 | Î | 11001110 | Ⅰ Ⅱ Ⅴ Ⅵ Ⅶ | 242 | ò | 11110010 | Ⅰ Ⅱ Ⅲ Ⅳ Ⅶ |
| 207 | Ï | 11001111 | Ⅰ Ⅱ Ⅴ Ⅵ Ⅶ Ⅷ | 243 | ó | 11110011 | Ⅰ Ⅱ Ⅲ Ⅳ Ⅶ Ⅷ |
| 208 | Ð | 11010000 | Ⅰ Ⅱ Ⅳ | 244 | ô | 11110100 | Ⅰ Ⅱ Ⅲ Ⅳ Ⅵ |
| 209 | Ñ | 11010001 | Ⅰ Ⅱ Ⅳ Ⅷ | 245 | õ | 11110101 | Ⅰ Ⅱ Ⅲ Ⅳ Ⅵ Ⅷ |
| 210 | Ò | 11010010 | Ⅰ Ⅱ Ⅳ Ⅶ | 246 | ö | 11110110 | Ⅰ Ⅱ Ⅲ Ⅳ Ⅵ Ⅶ |
| 211 | Ó | 11010011 | Ⅰ Ⅱ Ⅳ Ⅶ Ⅷ | 247 | ÷ | 11110111 | Ⅰ Ⅱ Ⅲ Ⅳ Ⅵ Ⅶ Ⅷ |
| 212 | Ô | 11010100 | Ⅰ Ⅱ Ⅳ Ⅵ | 248 | ø | 11111000 | Ⅰ Ⅱ Ⅲ Ⅳ Ⅴ |
| 213 | Õ | 11010101 | Ⅰ Ⅱ Ⅳ Ⅵ Ⅷ | 249 | ù | 11111001 | Ⅰ Ⅱ Ⅲ Ⅳ Ⅴ Ⅷ |
| 214 | Ö | 11010110 | Ⅰ Ⅱ Ⅳ Ⅵ Ⅶ | 250 | ú | 11111010 | Ⅰ Ⅱ Ⅲ Ⅳ Ⅴ Ⅶ |
| 215 | × | 11010111 | Ⅰ Ⅱ Ⅳ Ⅵ Ⅶ Ⅷ | 251 | û | 11111011 | Ⅰ Ⅱ Ⅲ Ⅳ Ⅴ Ⅶ Ⅷ |
| 216 | Ø | 11011000 | Ⅰ Ⅱ Ⅳ Ⅴ | 252 | ü | 11111100 | Ⅰ Ⅱ Ⅲ Ⅳ Ⅴ Ⅵ |
| 217 | Ù | 11011001 | Ⅰ Ⅱ Ⅳ Ⅴ Ⅷ | 253 | ý | 11111101 | Ⅰ Ⅱ Ⅲ Ⅳ Ⅴ Ⅵ Ⅷ |
| 218 | Ú | 11011010 | Ⅰ Ⅱ Ⅳ Ⅴ Ⅶ | 254 | þ | 11111110 | Ⅰ Ⅱ Ⅲ Ⅳ Ⅴ Ⅵ Ⅶ |
| 219 | Û | 11011011 | Ⅰ Ⅱ Ⅳ Ⅴ Ⅶ Ⅷ | 255 | ÿ | 11111111 | Ⅰ Ⅱ Ⅲ Ⅳ Ⅴ Ⅵ Ⅶ Ⅷ |
| 220 | Ü | 11011100 | Ⅰ Ⅱ Ⅳ Ⅴ Ⅵ |  | | | |
| 221 | Ý | 11011101 | Ⅰ Ⅱ Ⅳ Ⅴ Ⅵ Ⅷ |  |  |  |  |
| 222 | Þ | 11011110 | Ⅰ Ⅱ Ⅳ Ⅴ Ⅵ Ⅶ |  |  |  |  |
| 223 | ß | 11011111 | Ⅰ Ⅱ Ⅳ Ⅴ Ⅵ Ⅶ Ⅷ |  |  |  |  |
| 224 | à | 11100000 | Ⅰ Ⅱ Ⅲ |  |  |  |  |
| 225 | á | 11100001 | Ⅰ Ⅱ Ⅲ Ⅷ |  |  |  |  |
| 226 | â | 11100010 | Ⅰ Ⅱ Ⅲ Ⅶ |  |  |  |  |
| 227 | ã | 11100011 | Ⅰ Ⅱ Ⅲ Ⅶ Ⅷ |  |  |  |  |
| 228 | ä | 11100100 | Ⅰ Ⅱ Ⅲ Ⅵ |  |  |  |  |
| 229 | å | 11100101 | Ⅰ Ⅱ Ⅲ Ⅵ Ⅷ |  |  |  |  |
| 230 | æ | 11100110 | Ⅰ Ⅱ Ⅲ Ⅵ Ⅶ |  |  |  |  |
| 231 | ç | 11100111 | Ⅰ Ⅱ Ⅲ Ⅵ Ⅶ Ⅷ |  |  |  |  |
| 232 | è | 11101000 | Ⅰ Ⅱ Ⅲ Ⅴ |  |  |  |  |
| 233 | é | 11101001 | Ⅰ Ⅱ Ⅲ Ⅴ Ⅷ |  |  |  |  |

**Note:** Ⅰ, Ⅱ, Ⅲ, Ⅳ, Ⅴ, Ⅵ, Ⅶ, and Ⅷ correspond to tag1, tag2, tag3, tag4, tag5, tag6, tag7, and tag8, respectively.

**Table S6.** Performance comparison of DTBs with other non-sequence DNA encoding strategies

| **Category** | **Time-resolved encoding** | **Structure encoding** | **Mass-tag encoding** | **Nanopore-current encoding** |
| --- | --- | --- | --- | --- |
| **Reference** | This work | Chu et al., 2026^[1]^ | Zhang et al., 2024^[2]^ | Chen et al., 2020^[3]^ |
| **Encoding carrier** | DTB | DNA condensate | MNTs-TDOF | DNA-HD |
| **Encoding dimension** | retention time and fluorescence | spatial distribution of fluorescence | m/z signals and relative intensities | nanopore current blockade peaks |
| **Encoding capacity** | 4⁸ = 65,536 | 3^4^ = 81 | 6^4^ = 1296 | 2^8^ = 256 |
| **Encoding density** | 1 bit/ carrier;  3.60 × 10^-2^ bits/base | 6.34 bits/ carrier;  ~ 6.34  bits/μm³ | 10.34 bits/ carrier;  ~3.34 × 10^5^  bits/μm³ | 8 bits/ carrier;  ~1.11 × 10^-3^ bits/base |
| **Readout method** | HPLC | CLSM | MALDI-MS | solid-state nanopore |
| **Readout speed** | 0.33 bits/min | ~6.34 bits/min | ~10.34 bits/min | ~1.60 bits/min |
| **Cost efficiency** | 2.08 bits/$ | ~6.47 bits/$ | ~6.50 bits/$ | ~3.26 bits/$ |

**Note：**

(i) All encoding density values refer to logical coding density rather than actual physical storage density.

(ii) For the DTB system, the readout speed and cost efficiency were estimated from the single-channel HPLC readout used in this work, where one chromatogram contains 8 bits of information and requires 24 min for analysis, giving a readout speed of 0.33 bits/min. The encoding cost was estimated from the commercial synthesis cost of FAM-labelled DNA tags (7.95 $/nmol), a DNA tag consumption of 0.01 nmol for each “1” bit, and an equal probability of “0” and “1” in random binary information. The readout cost was estimated from an HPLC usage fee of 0.147 $/min. Under these assumptions, the total encoding–readout cost was calculated as 7.95 $/nmol × 0.01 nmol × 0.5 + (24 min × 0.147 $/min) / 8 bits =0.481 $/bit, corresponding to an overall cost efficiency of 2.08 bits/$.

(iii) For structure-based, mass-tag, and nanopore-current encoding systems, the readout speeds and cost efficiencies are approximate estimates based on the available information and stated assumptions.

(iv) Abbreviations: CLSM, confocal laser scanning microscopy; MNTs-TDOF, Mass Nanotags–Tetrahedral DNA Origami Frames; MALDI-MS, matrix-assisted laser desorption/ionization mass spectrometry; DNA-HD, DNA hard drive.

**Table S7.** DNA sequences used in the design and optimization of DNA Temporal Tags.

|  | **Name** | **Sequence 5’→3’/ Int modifications** | **5’modification** | **3’modification** |
| --- | --- | --- | --- | --- |
| **Vary modification type** | OH-T_20_-FAM | TTTTTTTTTTTTTTTTTTTT | OH | FAM |
|  | Pho-T_20_-FAM | TTTTTTTTTTTTTTTTTTTT | Phosphorylation | FAM |
|  | Acr-T_20_-FAM | TTTTTTTTTTTTTTTTTTTT | Acrydite | FAM |
|  | Mb-T_20_-FAM | TTTTTTTTTTTTTTTTTTTT | Methylene blue | FAM |
|  | NH_2_-T_20_-FAM | TTTTTTTTTTTTTTTTTTTT | NH_2_ C12 | FAM |
|  | Fer-T_20_-FAM | TTTTTTTTTTTTTTTTTTTT | Ferrocene | FAM |
|  | Dig-T_20_-FAM | TTTTTTTTTTTTTTTTTTTT | Digoxigenin | FAM |
|  | Pyr-T_20_-FAM | TTTTTTTTTTTTTTTTTTTT | Pyrene | FAM |
|  | Bio-T_20_-FAM | TTTTTTTTTTTTTTTTTTTT | Biotin | FAM |
|  | C18-T_20_-FAM | TTTTTTTTTTTTTTTTTTTT | C18 | FAM |
|  | Pyr-Pyr-T_20_-FAM | /iPyrene/TTTTTTTTTTTTTTTTTTTT | Pyrene | FAM |
|  | Acr-Pyr-T_20_-FAM | /iPyrene/TTTTTTTTTTTTTTTTTTTT | Acrydite | FAM |
|  | Pyr-T_10_-Pyr-T_10_-FAM | TTTTTTTTTT/iPyrene/TTTTTTTTTT | Pyrene | FAM |
|  | Acr-T_10_-Pyr-T_10_-FAM | TTTTTTTTTT/iPyrene/TTTTTTTTTT | Acrydite | FAM |
| **Vary strand length** | Pyr-T_62_-FAM | TTTTTTTTTTTTTTTTTTTTTTTTTTTTTTTTTTTTTTTTTTTTTTTTTTTTTTTTTTTTTT | Pyrene | FAM |
|  | Pyr-T_48_-FAM | TTTTTTTTTTTTTTTTTTTTTTTTTTTTTTTTTTTTTTTTTTTTTTTT | Pyrene | FAM |
|  | Pyr-T_34_-FAM | TTTTTTTTTTTTTTTTTTTTTTTTTTTTTTTTTT | Pyrene | FAM |
|  | Pyr-T_6_-FAM | TTTTTT | Pyrene | FAM |
|  | Fer-T_62_-FAM | TTTTTTTTTTTTTTTTTTTTTTTTTTTTTTTTTTTTTTTTTTTTTTTTTTTTTTTTTTTTTT | Ferrocene | FAM |
|  | Fer-T_48_-FAM | TTTTTTTTTTTTTTTTTTTTTTTTTTTTTTTTTTTTTTTTTTTTTTTT | Ferrocene | FAM |
|  | Fer-T_34_-FAM | TTTTTTTTTTTTTTTTTTTTTTTTTTTTTTTTTT | Ferrocene | FAM |
| **Vary base type** | Pyr-A_20_-FAM | AAAAAAAAAAAAAAAAAAAA | Pyrene | FAM |
|  | Pyr-C_20_-FAM | CCCCCCCCCCCCCCCCCCCC | Pyrene | FAM |
|  | Pyr-(G_4_A)_4_-FAM | GGGGAGGGGAGGGGAGGGGA | Pyrene | FAM |

**Table S8.** DNA sequences used in the information encoding system.

|  | **Name** | **Sequence 5’→3’/ Int modifications** | **5’modification** | **3’modification** |
| --- | --- | --- | --- | --- |
| **FAM fluorescence** | tag1 | CGTGTGGTTTT | Phosphorylation | FAM |
|  | tag2 | TTTTAGAGGATCGTGTGGTTTT | OH | FAM |
|  | tag3 | TTTTAGAGGATCGTGTGGTTTT | NH_2_ C12 | FAM |
|  | tag4 | TTTTAGAGGATCGTGTGGTTTT | Digoxigenin | FAM |
|  | tag5 | TTTTTTTTTTTTTTTTTTTTTTTTTTTTTTTTTTTTTTTTTTTTTTTTTTTTTTAGAGGATCGTGTGGTTTT | Pyrene | FAM |
|  | tag6 | T/iPyrene/TTTTAGAGGATCGTGTGGTTTT | Acrydite | FAM |
|  | tag7 | TTTTAGAGGATCGTGTGGTTTT | Pyrene | FAM |
|  | tag8 | TT/iPyrene/TTTTTTTTTTTTTTTTTTTTTTTTTTTTTTTTTTTTTAGAGGATCGTGTGGTTTT | Pyrene | FAM |
| **AF647 fluorescence** | tag1 | CGTGTGGTTTT | Phosphorylation | AF647 |
|  | tag2 | TTTTAGAGGATCGTGTGGTTTT | Acrydite | AF647 |
|  | tag3 | TTTTAGAGGATCGTGTGGTTTT | NH_2_ C12 | AF647 |
|  | tag4 | TTTTAGAGGATCGTGTGGTTTT | Digoxigenin | AF647 |
|  | tag5 | TTTTTTTTTTTTTTTTTTTTTTTTTTTTTTTTTTTTTTTTTTTTTTTTTTTTTTAGAGGATCGTGTGGTTTT | Pyrene | AF647 |
|  | tag6 | T/iPyrene/TTTTAGAGGATCGTGTGGTTTT | Acrydite | AF647 |
|  | tag7 | TTTTAGAGGATCGTGTGGTTTT | Pyrene | AF647 |
|  | tag8 | TT/iPyrene/TTTTTTTTTTTTTTTTTTTTTTTTTTTTTTTTTTTTTAGAGGATCGTGTGGTTTT | Pyrene | AF647 |

**Table S9.** DNA sequences used in the information encryption system.

|  | **Name** | **Sequence 5’→3’/ Int modifications** | **5’modification** | **3’modification** |
| --- | --- | --- | --- | --- |
| **Public key** | tag1 | CGTGTGGTTTT | Phosphorylation | FAM |
|  | SDT2 | TTTTAGAGGAT | OH | —— |
|  | SDT3 | TTTTAGAGGAT | NH_2_ C12 | —— |
|  | SDT4 | TTTTAGAGGAT | Digoxigenin | —— |
|  | SDT5 | TTTTTTTTTTTTTTTTTTTTTTTTTTTTTTTTTTTTTTTTTTTTTTTTTTTTTTAGAGGAT | Pyrene | —— |
|  | SDT6 | T/iPyrene/TTTTAGAGGAT | Acrydite | —— |
|  | SDT7 | TTTTAGAGGAT | Pyrene | —— |
|  | SDT8 | TT/iPyrene/TTTTTTTTTTTTTTTTTTTTTTTTTTTTTTTTTTTTTAGAGGAT | Pyrene | —— |
| **Private key** | S* | AAAACCACACGATCCTCTAAAA | —— | —— |

**Reference**

[1] Chu, L., Wan, L., Wang, H., et al., “Hierarchical Core–Shell DNA Condensates Enable Programmable Information Storage and Encryption,” *Nature Communications* 17, no. 1 (2026): 401. <https://doi.org/10.1038/s41467-025-67093-w>.

[2] Zhang, X., Dong, Y., Wang, Y., et al., “Quality Control of Mass-Encoded Nanodevices by Compartmented DNA Origami Frames for Precision Information Coding and Logic Mapping,” *Angewandte Chemie International Edition* 136, no. 4 (2024): e202313446. <https://doi.org/10.1002/ange.202313446>.

[3] Chen, K., Zhu, J., Bošković, F., Keyser, U. F., “Nanopore-Based DNA Hard Drives for Rewritable and Secure Data Storage,” *Nano Letters* 20, no. 5 (2020): 3754–3760. <https://doi.org/10.1021/acs.nanolett.0c00755>.
